# Natural Variation at the *FRD3* MATE Transporter Locus Reveals Cross-Talk between Fe Homeostasis and Zn Tolerance in *Arabidopsis thaliana*

**DOI:** 10.1371/journal.pgen.1003120

**Published:** 2012-12-06

**Authors:** Christophe Pineau, Stéphanie Loubet, Cécile Lefoulon, Claude Chalies, Cécile Fizames, Benoit Lacombe, Marina Ferrand, Olivier Loudet, Pierre Berthomieu, Odile Richard

**Affiliations:** 1 CNRS-INRA-MontpellierSupAgro-UM2, UMR Biochimie et Physiologie Moléculaire des Plantes, Montpellier, France; 2 INRA, UMR1318, Institut Jean-Pierre Bourgin, Versailles, France; University of Lausanne, Switzerland

## Abstract

Zinc (Zn) is essential for the optimal growth of plants but is toxic if present in excess, so Zn homeostasis needs to be finely tuned. Understanding Zn homeostasis mechanisms in plants will help in the development of innovative approaches for the phytoremediation of Zn-contaminated sites. In this study, Zn tolerance quantitative trait loci (QTL) were identified by analyzing differences in the Bay-0 and Shahdara accessions of *Arabidopsis thaliana*. Fine-scale mapping showed that a variant of the Fe homeostasis-related *FERRIC REDUCTASE DEFECTIVE3* (*FRD3*) gene, which encodes a multidrug and toxin efflux (MATE) transporter, is responsible for reduced Zn tolerance in *A. thaliana*. Allelic variation in *FRD3* revealed which amino acids are necessary for FRD3 function. In addition, the results of allele-specific expression assays in F1 individuals provide evidence for the existence of at least one putative metal-responsive *cis*-regulatory element. Our results suggest that FRD3 works as a multimer and is involved in loading Zn into xylem. Cross-homeostasis between Fe and Zn therefore appears to be important for Zn tolerance in *A. thaliana* with FRD3 acting as an essential regulator.

## Author Summary

Plants are adapted to soils in which the amounts of different nutrients vary widely, like Zn-deficient or Zn-contaminated soils. Exploring the molecular bases of plant adaptation to Zn-contaminated soils is important in determining strategies for phytoremediation. Here, we describe the mapping and characterization of a QTL for Zn tolerance in *A. thaliana* that underlies the natural variation of the root response to excess Zn. This physiological variation is controlled by different alleles of the *AtFRD3* gene, which codes for a citrate transporter that uploads citrate into the xylem sap, hence playing a role in Fe homeostasis. In the Zn-sensitive accession Shahdara, the expression of *AtFRD3* is drastically reduced and the protein encoded is unable to efflux citrate *in vitro*. Less Fe and Zn are found in Shahdara root exudates, and less Fe and Zn are translocated from root to shoot when Zn is in excess. We deduce that a fine-tuned Fe and Zn homeostasis is crucial for Zn tolerance in *A. thaliana*. Finally, as a range of alleles were identified, some rare, it was possible to define a sequence motif that is a putative metal-responsive *cis*-element and demonstrate that two amino acids are essential for the function of the FRD3 transporter.

## Introduction

Zinc (Zn) is an essential micronutrient for a wide range of cellular functions [1], yet if present in excess, it causes drastic toxicity symptoms resulting in yield reduction and stunted growth [1]. Soil mineral nutrient content varies greatly and plants have adapted to such varied environments in different ways. In highly Zn-contaminated soils only a small range of plants can develop. These Zn-tolerant plants may also hyper-accumulate Zn, like *Arabidopsis halleri* and *Noccaea caerulescens* [2]. For phytoremediation, for instance replanting in Zn-contaminated soils, it is crucial to understand the molecular bases of such adaptative processes. *A. halleri* and *N. caerulescens* have received extensive attention and various Zn transporters, such as HMA4 and MTP1, and the Zn chelator nicotianamine were shown to be important for Zn tolerance and accumulation [2]–[6]. However, genetic approaches are not straightforward in these species, mainly because of the lack of available molecular tools, so it may be difficult to find additional genes involved in Zn tolerance in these species.

To identify new genes involved in Zn tolerance, an alternative genetic approach is to benefit from how one species, *Arabidopsis thaliana*, has adapted to the environment and characterize the genetic factors underlying this natural variation. In *A. thaliana*, the numerous available natural accessions and RIL populations facilitate traditional linkage analyses and genome wide association studies. In particular QTL mapping analyses have already been successful in identifying genetic factors controlling metal tolerance such as aluminum and copper tolerance [7], [8]. In *A. thaliana*, the natural variation in Zn accumulation in different organs and growth conditions has been characterized [9]–[15]. Although no Zn tolerance mechanism has been identified yet, *A. thaliana* accessions display substantial natural variation in their response to excess Zn [16].

In the presence of an excess of a given mineral element in the medium, from a nutritional point of view, there is some competition between this element and related elements; plants need to coordinate homeostasis to avoid ion imbalances. For instance, in *A. thaliana*, in the presence of an excess of Zn, plants reduce iron (Fe) accumulation in shoots becoming prone to Fe-deficiency [17]. In *A. halleri*, a fine regulation of Fe homeostasis contributes to Zn tolerance [17]. Many studies have revealed the importance of the coordination between homeostatic mechanisms for different metals, but the identification of the corresponding molecular components is rare [18].

Here, we describe the mapping of a QTL for the Zn root response which led to the identification of the *AtFRD3* gene, which plays a role in Fe nutrition [19], as being responsible for the natural variation in the root response to excess of Zn between the Bay-0 and Shahdara *A. thaliana* accessions. Results of this study confirmed the importance of the coordination between Fe and Zn homeostasis for the tolerance to Zn excess, with FRD3 being a major regulator of the cross homeostasis.

## Results/Discussion

### Identification of Zn tolerance QTLs in *A. thaliana*

**Figure 1:**
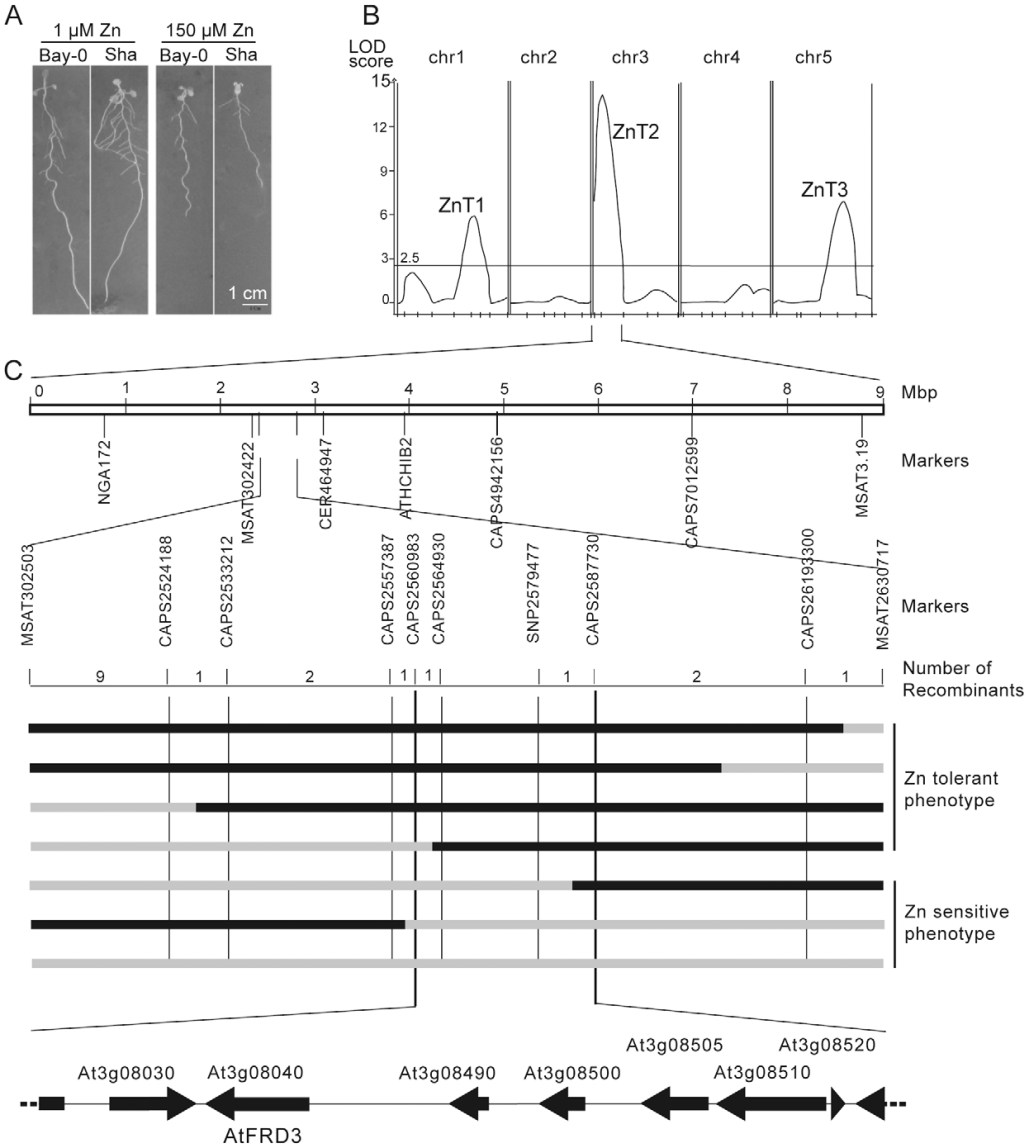
QTL and fine-mapping of the ZnT2 locus. (A) Root phenotype of 10-day-old Bay-0 and Sha plants grown on agar plates containing 1 µM or 150 µM Zn. Bay-0 is more Zn-tolerant than Sha. Scale bar, 1 cm. (B) QTL map for the response of the primary root length to Zn (relative primary root length of plants grown at 150 µM and 1 µM Zn), evaluated in a sub-set of 165 RILs from the Bay-0×Sha RIL population. The map was obtained by composite interval mapping and a LOD threshold of 2.5 obtained from permutation. (C) Fine mapping of the ZnT2 QTL. ZnT2, initially mapped between markers NGA172 and MSAT3.19, was localized between MSAT302503 and MSAT2630717 and narrowed down to between CAPS2560983 and CAPS2587730 by recombinant screening. This 23 kb-long region encompasses 7 full-length genes including *FRD3*. Zn tolerance phenotypes of recombinant plants with informative genotypes are given. Black and grey bars represent Bay and Sha genomes, respectively.

To explore the molecular bases of Zn tolerance variation, a QTL analysis was performed in the Bay-0×Sha recombinant inbred line (RIL) population [20] where the trait considered was the relative primary root length (RelPR_150_) between high Zn (150 µM Zn) and low Zn (1 µM Zn) control conditions (Figure 1A, 1B, Table S1, Figure S1). Three QTLs named ZnT1, ZnT2, and ZnT3 were localized on chromosomes 1, 3 and 5, respectively. No epistasis was observed either between the identified QTLs or between the QTLs and other regions of the genome. The ZnT1 and ZnT2 QTLs were confirmed by analyzing relative primary root length in the progeny of heterogeneous inbred families in which either the Sha or Bay allele was fixed (Figure S2). ZnT2 was the main-effect QTL explaining 29% of the total phenotypic variation (Table S1); the recessive Sha allele (Figure S3) was responsible for the greater reduction in primary root length observed in response to 150 µM Zn compared to the Bay-0 (Bay) allele (Table S1).

### *AtFRD3* underlies natural variation in root response to excess of Zn

A mapping population of 2,296 plants derived from a near-isogenic line (NIL) bearing a Sha introgression at the ZnT2 QTL in an otherwise Bay background (NIL[Sha]; Figure S4) was analyzed and ZnT2 was mapped to a 23-kb region spanning 7 genes (Figure 1C). Among these genes, the *FERRIC REDUCTASE DEFECTIVE3* (*FRD3*) gene was considered a good micronutrient-related candidate as it encodes a citrate efflux transporter involved in Fe nutrition [19], [21]–[23].

**Figure 2:**
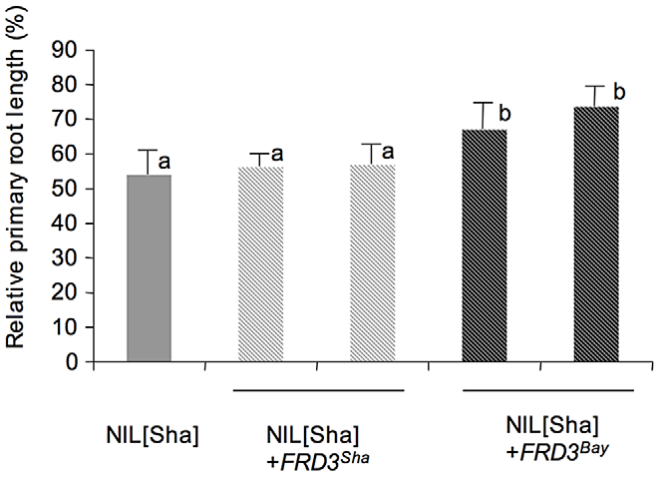
Functional complementation. Relative primary root lengths are shown for NIL[Sha] plants and for transgenic NIL[Sha] plants expressing either the *FRD3^Bay^* or the *FRD3^Sha^* allele. Relative primary root length is the primary root length of plants grown at 150 µM Zn as a percentage of the primary root length of plants grown at 1 µM Zn. Error bars represent confidence intervals calculated after logarithmic transformation of data [41] (P<0.05; n = 7 to 21). Similar increased relative root lengths were observed in 2 additional independent NIL[Sha] lines complemented with *FRD3^Bay^* (data not shown).

To confirm a functional role of *FRD3* in the Zn response by genetic complementation, wild-type Col-0 and the *frd3*-*7* knock-out mutant were both crossed separately with Bay-0 and NIL[Sha] plants and the primary root lengths of F1 progeny were measured. The primary root growth of NIL[Sha], inhibited in the presence of 150 µM Zn, was complemented with the *FRD3^Col^* allele but not with the *frd3*-*7* mutant allele. The primary root length of Bay-0 was little affected by the presence of either allele. This indicates that the ZnT2 QTL is likely to be allelic to the *FRD3* candidate gene (Figure S1, Figure S5). Independently, the dominant *FRD3^Bay^* allele was introduced into NIL[Sha] plants by genetic transformation. This resulted in the conversion of the recessive Zn-sensitive phenotype of NIL[Sha] plants into a more Zn-tolerant phenotype (Figure 2). Together, these two approaches demonstrate that *FRD3* has a functional role in determining the Zn-responsive ZnT2 trait.

### Modifications in *AtFRD3* explain variation in gene expression and transport activity

**Figure 3:**
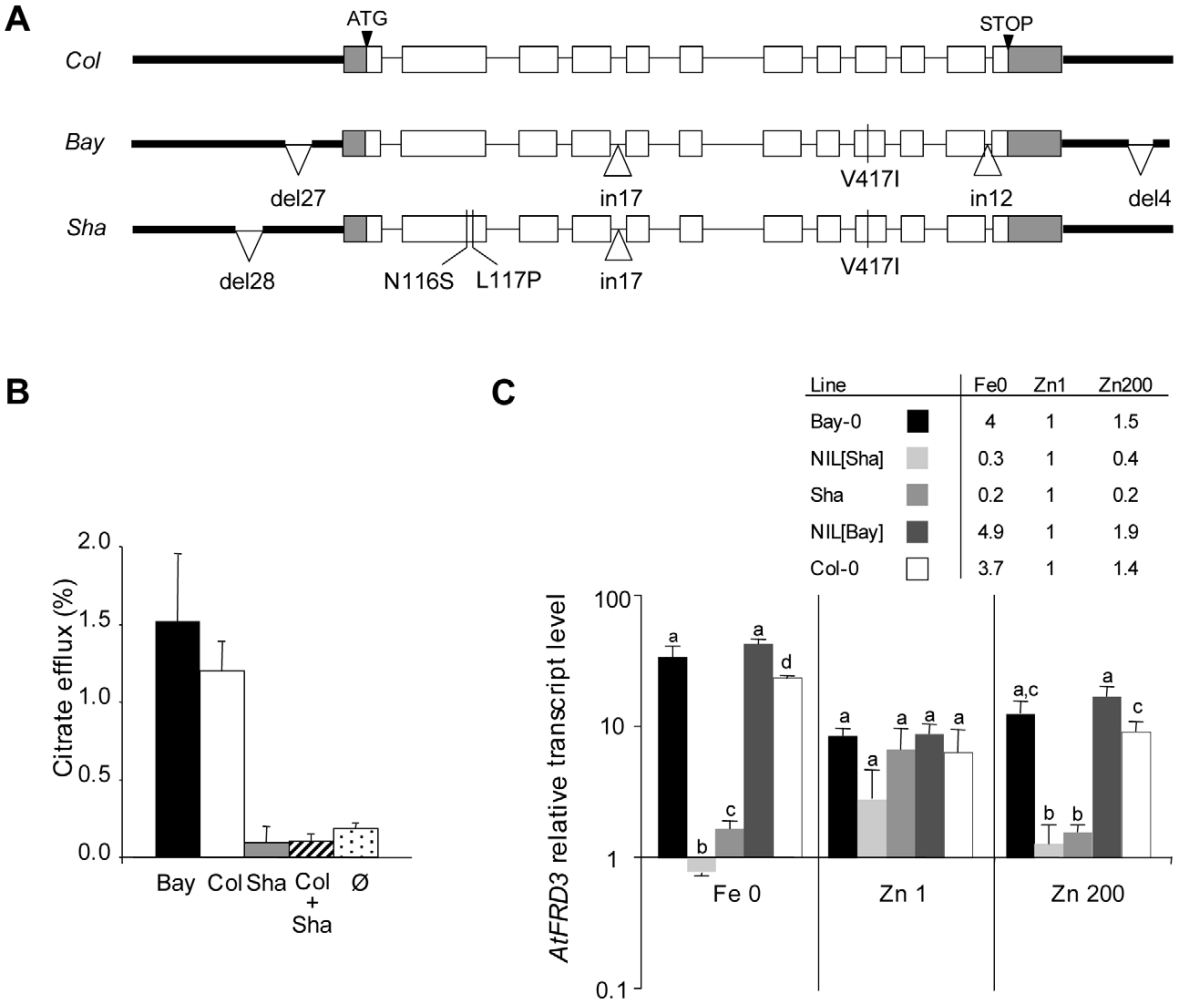
Natural variation in the *FRD3* sequence, FRD3 transport activity, and *FRD3* transcript accumulation. (A) Gene organization and allelic variations identified at the *FRD3* locus in Col-0 (reference sequence), Bay-0 and Sha. In non-coding regions, only insertions and deletions are shown. In the coding region, synonymous SNPs are not indicated. (B) ^13^[C]-citrate efflux activity resulting from the expression of FRD3^Bay^ (Bay), FRD3^Col^ (Col) and FRD3^Sha^ (Sha) proteins or the co-expression of FRD3^Sha^ and FRD3^Col^ (Col+Sha) in *Xenopus* oocytes. In control oocytes (Ø), no RNA was injected. Data are the means ± S.D.M. for n = 3 sets of 14 oocytes. (C) *FRD3* transcript accumulation under Fe deficiency (Fe 0), control conditions (Zn 1) and excess Zn (Zn 200) in parental and NIL lines. *FRD3* transcript levels are shown relative to the transcript level of ACT2/ACT8. Values represent the means ± S.D.M. for n = 3 biologically independent experiments. Different letters above bars indicate significantly different values within a treatment set (P<0.05) according to a non-parametric test for Fe0, and Zn200 and a Tukey test for Zn1. The table indicates *FRD3* transcript levels for the Fe0 and Zn200 conditions relative to those for the control Zn1 condition.

Sequencing the *FRD3* gene in the Bay-0 and Shahdara accessions revealed differences due to multiple nucleotide substitutions, deletions and insertions (Figure 3A, Figure S6). The main variations differentiating the *FRD3^Bay^* and *FRD3^Sha^* alleles were 27-bp and 28-bp indels in the promoter region, two non-synonymous substitutions in exon 2 in the *FRD3^Sha^* allele and a 12-bp indel in the last intron. Other differences were a 4-bp indel in the 3′ UTR, ten 1- or 2-bp indels in non-coding regions, 5 synonymous SNPs in coding regions and 48 SNPs in non-coding regions. The non-synonymous substitutions in exon 2 induce N116S and L117P substitutions in the FRD3^Sha^ polypeptide compared to FRD3^Bay^ and FRD3^Col^ polypeptides. Based on the analysis of the five main allelic variations in 109 *A. thaliana* accessions, five haplotypes were found (Figure S7, Table S2). Interestingly, the 28-bp deletion present in the promoter and the two non-synonymous substitutions in exon 2 were always associated with each other and this haplotype was found in 6% of the 109 accessions (Figure S7). No haplotype was found in which the promoter deletion and non-synonymous substitutions were separate. We hypothesized that the 28-bp deletion present in the promoter and/or the two non-synonymous substitutions present in exon 2, as found in the *FRD3^Sha^* allele, are the allelic variations responsible for the Zn-sensitive phenotype. A review of the types of nucleotide polymorphisms that underlie QTLs revealed that such a combination of nucleotide polymorphism in both promoter and coding regions, altering both gene expression and protein function, is not rare [24].

AtFRD3 is reported to be a citrate efflux transporter [22], so the functionality of the different protein variants was tested using a citrate efflux assay in *Xenopus* oocytes. The FRD3^Bay^ or FRD3^Col^ proteins were found to mediate citrate efflux, but the N116S and L117P substitutions abrogated this function in the FRD3^Sha^ protein (Figure 3B). The *FRD3^Bay^* and *FRD3^Sha^* genes are also expressed differently. Expression of *FRD3^Bay^* was induced by an excess of Zn and by Fe shortage (FRD3 plays a role in Fe deficient conditions [19], [21]–[23]), while *FRD3^Sha^* was expressed at a markedly lower level under the same conditions (Figure 3C). Genotype-dependent expression of *FRD3* under Fe deficiency has already been observed in natural accessions [25]. Thus the *FRD3^Sha^* gene, the Zn-sensitive allele, not only encodes a non-functional transporter but is also weakly expressed in the presence of high concentrations of Zn in the medium.

FRD3 belongs to the MATE family, one of the multidrug transporter families encountered in all living organisms [26]. Little is known of how MATE transporters work. When FRD3^Col^ was co-expressed with FRD3^Sha^ in *Xenopus* oocytes the ability of the FRD3^Col^ protein to efflux citrate was completely lost (Figure 3B). This would suggest first that FRD3^Sha^ interacts with FRD3^Col^, indicating that the FRD3 transporter functions as a multimer, and second that FRD3^Sha^ is a dominant negative isoform. The discovery that N116 and L117 are crucial for FRD3 function and that FRD3 likely functions as a multimer is thus an important step in understanding the general mode of action of MATE transporters.

The apparent dominant negative effect of FRD3^Sha^ in the citrate efflux assay in *Xenopus* oocytes may seem inconsistent with the observed recessive character of the Zn-sensitive phenotype associated with the *FRD3^Sha^* allele *in planta*. Compared to the control condition, transcript levels of the *FRD3^Sha^* allele are 2.5 to 5 times lower in response to Zn excess, while transcript levels of the *FRD3^Bay^* allele are 1.5 to 2 times higher in the same condition (Figure 3C). We therefore favor the hypothesis that *in planta*, the effect of down regulation of the *FRD3^Sha^* transcript predominates over the dominant negative effect of the FRD3^Sha^ protein. On this point, no accession harboring only the non-synonymous substitutions leading to the non-functional FRD3^Sha^ isoform was found after screening 109 *A. thaliana* accessions. This suggests that these mutations are strongly selected against and that a mutation that reduces transcription is needed to overcome the dominant negative effect of the non-synonymous substitutions.

### Towards the identification of a *cis*-regulatory element that controls *FRD3* induction in response to Fe deficiency

*FRD3* transcript accumulation was compared in NILs and natural accessions in relation to the haplotype. While *FRD3* expression was induced by Fe deficiency and by Zn excess in Bay-0, NIL[Bay] and Col-0, regulation occurred in the opposite sense in Sha and NIL[Sha] (Figure 3C). When correlated to the presence of certain polymorphisms in the gene particularly within the *FRD3* promoter, the results point to the likely existence of local Zn- and Fe-responsive, possibly *cis*-acting, regulatory elements (Figure 3C, Figure S7). The differential induction of *FRD3^Bay^* and *FRD3^Sha^* transcripts by Fe deficiency was tested through allele-specific expression assays in F1 individuals. The differential induction was maintained in the presence of the contrasting allele indicating that the response is controlled in *cis* (Figure S8A). The *cis*-acting differential regulation was confirmed in a cross between Ct-1 and Ita-0 (Figure S8B), where Ct-1 is a Bay-like accession in terms of *FRD3* transcriptional induction and sequence (Table S2), and Ita-0 only differs from Ct-1 at the 27bp-polymorphism (Figure S7) and is not transcriptionally responsive to Fe deficiency. Therefore, although we cannot totally exclude the possibility that polymorphisms outside the sequenced region play a role, the indel sequences identified are probably important. None of the known Zn- or Fe-responsive *cis*-elements from plants is present in the Bay or Col alleles of the *FRD3* promoter. The region around the 27-bp sequence in the *FRD3* promoter region may therefore represent a new type of metal-responsive *cis*-regulatory element.

### Impact of the FRD3 polymorphisms in Bay-0 and Shahdara on Fe nutrition

**Figure 4:**
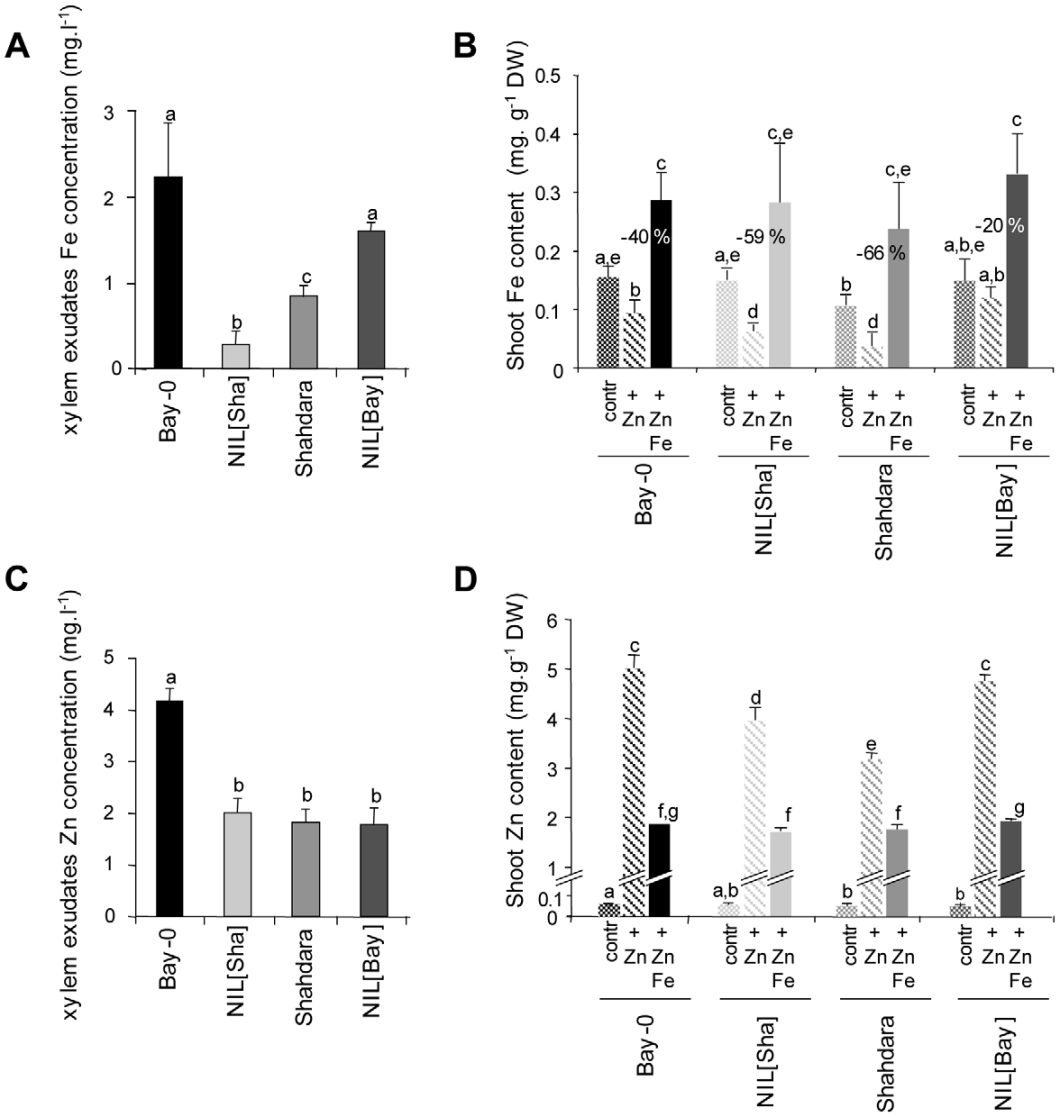
Fe and Zn homeostasis in NILs and parental lines. Fe (A) and Zn (C) concentrations in xylem exudates. Plants were grown in a growth chamber for 6 weeks and xylem exudates were collected during 30 min after removing aerial parts of the plant at the hypocotyl. Fe (B) and Zn (D) shoot content. Plants were grown on agar plates under control (contr.), 200 µM Zn (+ Zn), and 200 µM Fe plus 200 µM Zn (+ Zn Fe) conditions for 10 days. Percentages given above bars in (B) refer to the difference in shoot Fe content in ‘+ Zn’ conditions as a percentage of the control value. Measurements were performed on sets of 5 to 20 plants. Values are the means ± S.D.M. Different letters above bars refer to significantly different values (P<0.05), according to non-parametric tests for (A), (B) and (D) and Tukey tests for (C) where n = 3–8.

*FRD3* is known to be involved in Fe homeostasis [19], [21]–[23]. More precisely, FRD3 releases citrate into the xylem so Fe can be solubilized, transported to the shoots and loaded into leaf cells. Variation at the *FRD3* locus might therefore be expected to affect the Fe content of xylem sap. As expected, the Fe concentration in xylem sap of Sha plants and NIL[Sha] plants, which have the non-functional *FRD3^Sha^* allele, was much lower than in plants harboring the *FRD3^Bay-0^* allele (Figure 4A). However, unlike *frd3* mutants in the Col-0 background [19], [23], [27], they were neither dwarf nor chlorotic, and under control conditions, the shoot and root Fe content was normal. Also no Fe overload was observed in the root stele of plants carrying the *FRD3^Sha^* allele (Figure 4B, Figure S9, Figure S10). No indication of Fe deficiency, such as increased mRNA levels or constitutive activity of the root ferric reductase oxidase FRO2, was detected in plants harboring the *FRD3^Sha^* allele grown in control conditions, in contrast to what has been observed in *frd3^Col^* mutants [19] (Figure S11, Figure S12). Xylem exudates of NIL[Sha] plants did not contain less citrate than Bay-0 plants (Figure S13).

Altogether these observations may suggest that *FRD3^Sha^* is a leaky allele of *FRD3*. Even an inefficient transport activity may be sufficient to avoid the severe phenotypes that are observed with the *frd3* knock-out mutation. However, this hypothesis is not in agreement with the oocyte assay data showing that FRD3^Sha^ is not functional. An alternative hypothesis could be that *FRD3^Sha^* is a strong mutant allele and that another mechanism is responsible for loading citrate or another Fe-chelating agent into the xylem to compensate for the lack of FRD3-driven citrate delivery in Shahdara and Bay-0. To test the latter hypothesis, F2 plants issuing from a cross between Shahdara and *frd3-7* (KO mutant in Col-0 background), were assayed for Fe overaccumulation in the root stele using Perls' stain as a rapid assay of the functionality of FRD3 [21]. A quarter of the F2 population showed Fe overload in the root stele, indicating that this trait was under the control of one recessive locus (Table S3). Importantly, some of the F2 plants showing Fe overaccumulation in the root stele were not homozygous for the *frd3* knock-out and two plants showing no Fe overload in the root stele were homozygous for the *frd3* knock-out (Table S3). This result means that it is unlikely that *FRD3^Sha^* is a leaky allele of *FRD3* and that a still uncharacterized mechanism can compensate, at least partially, for the lack of functionality of FRD3, and is thus also involved in the translocation of Fe and Zn ions from the roots to the shoot. This mechanism is present in Shahdara, but it is missing in Col-0, thus explaining why the *frd3* mutant phenotype is stronger in the Col-0 background than in the Shahdara background. This compensatory mechanism is also most likely active in the four other accessions that harbor the FRD3^Sha^ haplotype, two of which (Hiroshima and 9481B) being even more tolerant to Zn excess than Bay-0 (Table S2).

### Impact of FRD3 on Zn tolerance and homeostasis

We investigated whether FRD3 has a direct impact on Zn homeostasis. The Zn concentration in the xylem exudates of NIL[Sha] plants was lower than that of Bay-0 plants in control conditions (Figure 4C), indicating that FRD3 plays a role in the loading of Zn into the xylem. In addition, in the presence of high concentrations of Zn in the culture medium, shoots of NIL[Sha] plants contained less Zn than Bay-0 shoots (Figure 4D). This clearly indicated that FRD3 is involved in the translocation of Zn from the roots to the shoot in *A. thaliana*. The mechanism by which FRD3 is involved in Zn tolerance in roots is in contrast more difficult to infer from the data. A positive correlation between translocation of Zn from root to shoots and Zn tolerance in roots has already been established from the analysis of *heavy metal atpase 4* (*hma4*) mutant lines and *HMA4*-overexpressing lines [28]–[30]. The interpretation was that reducing Zn translocation from the roots to the shoot resulted in an increase in the Zn content in roots that would be the cause of an increased sensitivity to Zn in roots. This interpretation may not be of great help to interpret our data. Whatever the Zn concentration in the medium, the presence of the FRD3^Sha^ allele does not induce any increase in the Zn content in roots compared to the FRD3^Bay^ allele (Figure S9). It is possible that although the Zn content is similar in roots of plants harboring the FRD3^Sha^ or FRD3^Bay^ allele the Zn distribution is different within the roots and that this difference would results in a difference in the root sensitivity to Zn. An alternative hypothesis could however be that the impact of FRD3 on Zn tolerance results from an impairment in Fe homeostasis.

We therefore investigated the phenotypic relationship between Zn and Fe homeostasis. High concentrations of Zn in the medium induced a decrease in the shoot Fe content (Figure 4B), mimicking Fe limiting conditions (Figure 3C, Figure S11). Vice versa increasing the Fe concentration in the medium resulted in a decrease in shoot Zn content (Figure 4D). Similar data have already been reported [17]. In response to the Zn constraint, the shoot Fe content in plants harboring the *FRD3^Sha^* allele was more reduced than in plants harboring the *FRD3^Bay-0^* allele (Figure 4B). Also, in the presence of excess Zn, more *FRO2* (*Ferric Reduction Oxidase*) transcripts were expressed in NIL[Sha] plants than in Bay-0 plants (Figure S11), indicating that FRD3^Sha^ confers sensitivity to Zn-induced Fe deficiency. Therefore we have shown that FRD3 acts at an intersection between Fe and Zn nutrition.

As shown above, FRD3 controls the loading of both Zn and Fe into the xylem and these two metals appear to compete for root-to-shoot transport. This conclusion leads to the hypothesis that the primary reason for which FRD3 has an impact on Zn tolerance would be that excess Zn impairs Fe nutrition and that FRD3 is an important control point in the Zn-Fe relationship. This is in agreement with, and may explain, recent observations on how the cross-homeostasis between Fe and Zn deals with excess Zn in *A. halleri* and *A. thaliana* [17]. In particular, exposing *A. halleri* plants to high Zn concentrations induced neither a marked alteration in Fe root-to-shoot transport nor Fe deficiency [17], which can be related to the fact that *FRD3* expression is 45 times higher in *A. halleri* than in *A. thaliana* [31], and thus would not be a limiting factor in the transport of either Zn or Fe from the roots to the shoot.

In conclusion, we show that the cross-homeostasis between Fe and Zn is important in the *A. thaliana* response to excess Zn and that FRD3 is an essential regulator of this cross homeostasis. This new perspective on FRD3, including the potential multimeric topology of the FRD3 transporter and the presence of an Fe deficiency-responsive element in the promoter, provides clear directions for further study of how FRD3 contributes to regulating plant micronutrient status.

## Materials and Methods

### Plant materials and growth conditions

QTL were identified from 165 Bay-0×Shahdara RIL lines [20] and validated using the HIF004, HIF044 and HIF338 lines available from INRA Versailles Genomic Resource Centre (http://dbsgap.versailles.inra.fr/vnat/). NIL[Bay] and NIL[Sha] were obtained from RIL112 and RIL070 following three successive back-crosses with the Shahdara and Bay-0 parental lines, respectively. After each backcrossing step, lines were selected with 38 microsatellite markers [20]. At the end of the process, NIL[Bay] plants had a Bay allele at marker ATHCHIB2 and Sha alleles at the other 37 markers. The reverse was true for NIL[Sha] plants. Seeds of *frd3-7* plants were kindly provided by C. Curie (BPMP, Montpellier, France).

Zn tolerance phenotypes were determined as previously described [16]. Plants were grown in the presence of 1 µM ZnSO_4_ (control condition) and in the presence of 150 µM ZnSO_4_ (high Zn condition). For each genotype the primary root length was measured in both conditions to obtain the relative primary root length (RelPR150 = (PR150/PR1)×100%). For Fe-response assays, plants were grown on control medium for 7 days and transferred to either Fe-deficient (Fe 0) or Fe-sufficient (Fe 50) medium for 4 days before collecting roots for RNA preparation or ferric reductase assays. The Fe-deficient medium was control medium without NaFeEDTA but with 300 µM ferrozine.

For the sampling of xylem exudates and Perls staining, plants were grown in compost for 6 or 3 weeks respectively in a growth chamber (20°C, 180 µmol.m^−2^.s^−1^ and an 8-h light/16-h dark photoperiod). Xylem exudates were collected by removing rosettes with a scalpel then placing a glass capillary tube on the root after discarding the first droplet exuded. After 30 min or 2 h of sap collection, xylem exudates were placed on ice then stored frozen at −20°C.

### Citrate, Fe, and Zn measurements

The citrate content of xylem exudates (50 µl to 100 µl from the 2-h collection) was analyzed by high performance ionic chromatography (LC20, Ionex) using an IonPac AS11 column and a 1 mM to 22 mM NaOH gradient.

The Zn and Fe contents of xylem exudates (5 µl from the 30-min collection) were estimated by atomic absorption spectrophotometry using graphite tube atomizers GTA220 (Varian) with omega platform tubes for Zn and partitioned tubes for Fe. Zn concentration in plant tissues was assessed as previously described [16] and Fe concentration in plant tissues was measured by the absorbance of Fe^2+^-*o*-phenanthroline at 510 nM [32].

### QTL analysis and fine-mapping of ZnT2

Core-Pop165 plants from the Bay-0×Shahdara RIL population [20] were grown on agar plates as previously described [16]. Primary root lengths were determined for ten plants from each of control (1 µM ZnSO_4_; PR1) and excess Zn (150 µM ZnSO_4_; PR150) agar plates. Relative primary root length (RelPR150 = (PR150/PR1)×100%) was analyzed using QTLCartographer (http://statgen.ncsu.edu/qtlcart/). Composite interval mapping (CIM) was performed using model 6 and the LOD significance threshold was obtained from permutation analyses. The percentage of variance explained by each QTL and its predicted allelic effect were obtained from QTLCartographer.

Fine-mapping populations were obtained by crossing NIL[Sha] plants with Bay-0 plants, producing an F_1_ population which was self-pollinated to produce an F_2_ population. DNA was extracted from plants (grown on soil in the greenhouse) by freeze-drying and grinding cotyledons in 300 µl buffer (100 mM Tris HCl, 1.5 M NaCl, 20 mM EDTA, 2% (w/v) mixed alkyltrimethyl ammonium bromide (Sigma), 0.5% (w/v) sodium sulfite, 1% (v/v) PEG6000). After chloroform extraction, DNA was precipitated using isopropanol, dried then dissolved in 50 µl water. Fine-mapping was done in two steps; first 624 plants were screened for recombination between MSAT302422 and CAPS7012599, then 1,672 plants were screened for recombination between MSAT302503 and MSAT2630717. Markers used for genotyping are described by INRA Versailles Genomic Resource Centre (http://dbsgap.versailles.inra.fr/vnat/) or in Table S4, for those newly identified here.

### *FRD3* sequencing

*FRD3* alleles were amplified from genomic DNA of Bay-0 and Shahdara using KlenTaq LA DNA Polymerase Mix (#D5062, Sigma Aldrich) with primers promo1F, promo2F, exon2R, intron4F, exon12R and post3 (see Table S5 for primer sequences) and three independent PCR products were sequenced.

### Transgenic plants

The genomic sequences of *FRD3* including the promoter were amplified from Bay-0 and Shahdara genomic DNA (Table S5), cloned in the pGEM-T vector, verified by resequencing, then cloned in the pGREEN0179 vector [33]. Binary recombinant vectors were introduced into *Agrobacterium tumefaciens* strain GV3101, which was used to transform NIL[Sha] plants with either *FRD3^Bay^* or *FRD3^Sha^* by the floral dip method [34]. Transformants were selected on MS/2 agar plates with 50 mg L^−1^ hygromycin. Single T-DNA insertion and homozygous lines were successively selected during segregation analysis.

### Quantitative gene expression

RNA extraction, cDNA preparation and real-time quantitative RT–PCR were done as previously described [16]. Primers used are listed in Table S5. Three independent biological experiments were done to analyze transcript levels, once relative to ACT2/ACT8 [35] transcript levels and once relative to transcript levels of ACT2/ACT8 [35], clathrin [36] (At4g24550), At5g12240 [37] and PP2A [37] (At1g13320). Similar results were observed in the two technical replicates.

### Allele-specific expression assays

Two pairs of accessions were analyzed using different SNPs, Bay-0 versus Shahdara and Ct-1 versus Ita-0. Parents and F1 individuals (from reciprocal crosses) were grown *in vitro* as described above and transferred onto Fe-deficient media. RNA was extracted from roots and cDNA prepared as described above. Pyrosequencing reactions were set up around SNPs in the parental coding sequence of *FRD3* to assess the relative contribution of each allele to the mRNA population of mRNA [38]. Pyrosequencing was performed on F1 cDNA, on 1∶1 mixtures of parental cDNA, and on F1 genomic DNA as a control to normalize the ratios against possible pyrosequencing biases. Anything significantly driving allele-specific expression in hybrids is, by definition, acting in cis as F1 nuclei contain a mix of all trans factors [39]. In the Bay/Sha experiment, SNP1 and SNP2 interrogate ACA[A/G]GA[T/C]TGG with primers PyroSNP1-2_F and PyroSNP1-2_R-biotin for the PCR and PyroSNP1-2_Seq for the pyrosequencing reaction. In the Ct-1/Ita-0 experiment, SNP3 interrogates AA[C/T]GAT with primers PyroSNP3_F-biotin, PyroSNP3_R and PyroSNP3_Seq; and SNP4 interrogates TC[G/A]TTA with primers PyroSNP4_F, PyroSNP4_R-biotin and PyroSNP4_Seq (Table S5).

### Citrate efflux experiment

The predicted cDNAs of the different *FRD3* alleles (Figure 4, Figure S6) were obtained from *FRD3^Col^* cDNA (G14324; pENTR223_FRD3*^Col^*) by site-directed mutagenesis using the QuikChange Site-Directed Mutagenesis Kit (Stratagene, La Jolla, CA, USA). We obtained *FRD3^Bay^* cDNA by introducing a single base modification in exon 9 of *FRD3^Col^* cDNA and *FRD3^Sha^* cDNA by introducing the two SNPs in exon2 of *FRD3^Bay^* cDNA (see Table S5 for primer sequences). All mutated cDNAs were sequenced to verify the mutation(s) and the complete sequence. These cDNAs were transferred from pENTR to the pGEM-GWC vector by recombination using LR-clonase (Life Technologies, Grand Island, USA). The different *FRD3* cDNA clones were linearized with *Pst*I then transcribed using mMessage mMachine T7® Ultra Kit (Life Technologies). *Xenopus laevis* oocytes (CRBM, CNRS, Montpellier, France) were prepared as previously described [40] and injected with 20 ng of RNA coding for *FRD3^Col^*, *FRD3^Bay^* or *FRD3^Sha^* using a micropipette (10–15 µM tip diameter) and a pneumatic injector. Three days after injection, 14 oocytes (either injected with *FRD3* cRNAs or uninjected) were placed in modified Ringer solution (in which HEPES was replaced by 1 mM Tris) supplemented with 10 mM citrate. Each batch of oocytes was injected with 25 nl of 100 mM ^13^[C]-citrate (Sigma). After 5 min of recovery, oocytes were washed five times in 15 ml of cold modified Ringer solution (pH 6.5) and placed in 1 ml of modified Ringer buffer for efflux measurement. After 20 min, 100 µl of efflux buffer was sampled in three replicates and the ^13^[C] abundance (atom %) was analyzed by continuous-flow mass spectrometry using the Euro-EA Eurovector elemental analyzer coupled to an IsoPrime mass spectrometer (GV Instruments).

### Statistical analysis

Once the normality of residues had been tested, either one-way ANOVA and Tukey tests (for parametric comparison of means) or Kruskal-Wallis test followed by a non-parametric comparison of means were used. All these tests used an α-value of 0.05 and were done with the R software (using *shapiro*, *bartlett*, *aov*, *TukeyHSD*, *kruskal* and *nparcomp* functions of R; R Development Core Team). Two-way ANOVA was used to test the interaction between genotypes in F1 progeny using the *aov* function of R software. To test differences in relative primary root growth values, confidence intervals (α-value of 0.05) were calculated after *ln* transformation of data [41].

### Accession numbers

The genomic DNA sequences of *FRD3* reported in this paper have been deposited in the EMBL Nucleotide Sequence Database: HE803766 (Bay-0) and HE803767 (Shahdara).

## Supporting Information

Figure S1 Distribution of the primary root length phenotype among 165 RILs derived from the Bay-0×Shahdara RIL population. Root lengths are from 10-day-old plants grown on agar plates supplemented with 150 µM Zn (black) or not (grey). Mean primary root lengths of the Bay-0 and Shahdara parental lines are indicated.
(PDF)

Figure S2 Genotypes of the HIF044 (A), HIF004 and HIF338 (B) lines used to validate ZnT1 (C) and ZnT2 (D) QTLs respectively. HIF044, HIF004 and HIF338 are derived from RIL044, RIL004 and RIL338 that still segregate for the NGA128-MSAT1.13, MSAT302503-MSAT3.19 and NGA172-CAPS7012599 intervals, respectively. (A) (B) Red, green and black portions refer to Bay, Sha and heterozygote genotypes respectively. Horizontal bars represent the positions of the markers used for the genetic mapping of the Bay-0×Shahdara population [4]. The NGA128-MSAT1.13 genomic region includes the ZnT1 support interval and the overlapping MSAT302503-MSAT3.19 and NGA172-CAPS7012599 genomic regions include the ZnT2 support interval. (C) (D) From each of the three RILs, HIF progenies were produced that were fixed at the ZnT loci and thus harbored either the Sha or the Bay allele at these loci. Relative primary root length is the ratio between the primary root length of plants grown at 150 µM and 1 µM Zn respectively and is expressed as a percentage. Error bars represent confidence intervals calculated after a logarithmic transformation of data [41] (** indicates significant differences, P<0.05; n = 14 to 23).
(PDF)

Figure S3 Genetic dominance test at the ZnT2 locus. Relative primary root length is the primary root length of plants grown at 150 µM as a percentage of the primary root length of plants grown at 1 µM Zn. Error bars represent confidence intervals (*P*<0.05) calculated after a logarithmic transformation of data [41]; n = 9 to 19. Different letters above bars refer to significantly different relative primary root lengths (*P*<0.05). Reciprocal independent crosses were made between HIF004^Bay^ and HIF004^Sha^ and the Bay allele appeared to be dominant over the Sha allele in phenotypic tests (data not shown).
(PDF)

Figure S4 Genotypes of near isogenic lines NIL[Sha] and NIL[Bay]. NIL[Sha] and NIL[Bay] were obtained by back-crossing RIL070 and RIL112 to Bay-0 and Shahdara, respectively. Red and green bars refer to Bay and Sha genotypes respectively. Horizontal bars represent the position of the markers used for the genetic mapping of the RIL population [20]. Newly defined markers are described in Table S3.
(PDF)

Figure S5 Quantitative complementation test for ZnT2. Primary root length was measured in the presence of 150 µM Zn for F1 plants obtained from crosses of Bay-0 or NIL[Sha] plants with both the Col-0 wild type and *frd3.7* mutant plants. F1 plants were genotyped and phenotyped individually. Each value is the mean ± S.E.M., n = 16 to 19. The genotype interaction between the *FRD3.7* allele and ZnT2 is highly significant (*P*<0.001).
(PDF)

Figure S6 Alignment of the *AtFRD3* genomic sequences obtained from the Col-0, Bay-0 and Shahdara accessions of *A. thaliana*. Annotations refer to the Col-0 sequence. SNPs and indels are shaded. Black letters indicate intergenic and intronic sequences, red letters untranslated regions and green letters coding sequences. Start and stop codons are indicated in bold letters.
(PDF)

Figure S7 *AtFRD3* haplotypes in *A. thaliana* and *A. lyrata*. (A) *AtFRD3* gene structure. Narrow lines and bold lines represent untranscribed regions and introns respectively. Grey boxes and white boxes refer to untranslated and coding sequences respectively. (B) Five haplotypes were identified using the 5 markers (del28, del27, N116L, S117P, in12) in 109 accessions of *A. thaliana*. One accession representative of each haplotype is mentioned. The percentage of each haplotype among the 109 accessions is mentioned (%). The 109 accessions are listed in Table S2. The *A. lyrata* haplotype was deduced from Genbank sequence ADBK01000458.
(PDF)

Figure S8 Allele-specific expression assays of *FRD3* under Fe shortage. Ratio of allelic expression of Bay-0 vs Shahdara (A) and Ct-1 vs Ita-0 (B) in equal mixtures of parental cDNA and in F1 hybrid cDNA. Values are the mean ± S.E.M. of 6 to 10 ratios from at least three independent pyrosequencing replicates. Two different mRNA SNPs are interrogated per cross. Ratios of allelic expression in F1 hybrid plants are significantly different from 1 (for SNP1, *P*<4×10^−4^; for SNP2, *P*<7×10^−3^; for SNP3, *P*<1×10^−7^; for SNP4, *P*<1×10^−8^) and not significantly different from those in parental cDNA mixtures, indicating that this differential allelic expression is controlled in *cis*.
(PDF)

Figure S9 Fe and Zn homeostasis in NILs and parental lines. Fe (A) and Zn (B, C) root contents. Plants were grown on agar plates under control conditions (A, B) or under 200 µM Zn for 10 days before roots were harvested and the Fe and Zn content estimated. Measurements were performed on sets of 5 to 20 plants. Values are the mean ± S.D.M. where n = 4.
(PDF)

Figure S10 Localization of Fe in roots. Fe accumulation is visualized by Perls staining [21] of intact roots of plants grown on compost for 3 weeks. Only *frd3-7* plants accumulate Fe in the xylem. Scale bar, 50 µm.
(PDF)

Figure S11 *AtFRO2* transcript levels. *AtFRO2* (*FERRIC REDUCTION OXIDASE 2*) is an Fe deficiency marker. Its transcript levels were determined under Fe deficient (Fe0), control (Zn1) and excess Zn (Zn150) conditions in Bay-0 and Shahdara parental lines and in NILs. *FRO2* transcript levels are expressed relative to the transcript level of actin (ACT2/ACT8). Values are the means ± S.D.M. where n = 3 independent experiments.
(PDF)

Figure S12 Root ferric chelate reductase activity. Plants were grown on control medium for 7 days and transferred to either iron deficient (−Fe) or iron sufficient (+Fe) medium 4 days before the assay. Values are the mean ± S.D.M. where n = 3 sets of 3 plants. With the exception of *frd3-7*, all genotypes present a significantly different level of root ferric chelate reductase activity in roots subject to iron deficiency than is found in iron-sufficient treatments (*P*<0.05, Student test).
(PDF)

Figure S13 Citrate content of xylem exudates. Plants were grown in a growth chamber for 6 weeks and xylem exudates were collected during 2 h after removing aerial parts of the plant at the hypocotyl. Values are means ± S.E.M. where n = 8 to 12. Different letters above bars refer to significantly different values at *P*<0.05 according to a nonparametric test.
(PDF)

Table S1 QTL analyses. (a) Relative variation of the Primary Root length between 150 µM Zn and control condition (RelPR_150_). (b) The position of the QTL is expressed in cM from the 1st marker of the chromosome. (c) Name of the closest marker, according to the initial mapping approach. (d) Percentage of variance explained by the QTL. (e) Additive effect. Positive value indicates that for the three QTLs, the presence of the Sha allele at QTL decreases root length in the presence of Zn.
(PDF)

Table S2 Haplotypes and phenotypes of *A. thaliana* accessions genotyped at *FRD3*. (a) Representative haplotype according to Figure S7. (b) Inhibitory concentration 50 (µM) determined as the Zn concentration that reduced primary root length to 50% of control. Stars refer to data from reference [16]. (c) Accession number according to INRA Versailles Genomic Resource Centre (http://dbsgap.versailles.inra.fr/vnat/). (d) Accession number according to NASC, European Arabidopsis Stock Centre (http://arabidopsis.info).
(PDF)

Table S3 Segregation analysis of the Fe overaccumulation trait in the F2 plants issuing from a cross between Shahdara and *frd3-7*. (a) Genotype determined for the presence or absence of T-DNA using primers LBb1 and FRD3exon12R or FRD3exon7F and FRD3exon12R, respectively. (b) Fe overaccumulation is visualized by Perls' stain [21] of intact roots of plants grown on compost for 3 weeks. (c) Chi_2_ analysis of the segregation of Fe overaccumulation trait in the F2 progeny, H0 hypothesis was that this trait is under the control of one monogenic recessive locus.
(PDF)

Table S4 List of markers. (a) Position on chromosome 3 in bp. (b) CAPS for Cleaved Amplified Polymorphic Sequence; INDEL for insertion or deletion; MSAT for microsatellite; SNP for Single Nucleotide Polymorphism. (c) RE for Restriction Enzyme used for CAPS markers. (d) Markers used for the haplotyping of *AtFRD3* in accessions of *A. thaliana*.
(PDF)

Table S5 List of primers. (a) Present study. (b) ASE for Allele-Specific Expression.
(PDF)
