# Supplementary figures and images for: Natural Variation at the FRD3 MATE Transporter Locus Reveals Cross-Talk between Fe Homeostasis and Zn Tolerance in Arabidopsis thaliana
Source: PLoS Genet. 2012 Dec 6;8(12):e1003120. doi: 10.1371/journal.pgen.1003120 (PMC3516540; doi:10.1371/journal.pgen.1003120)

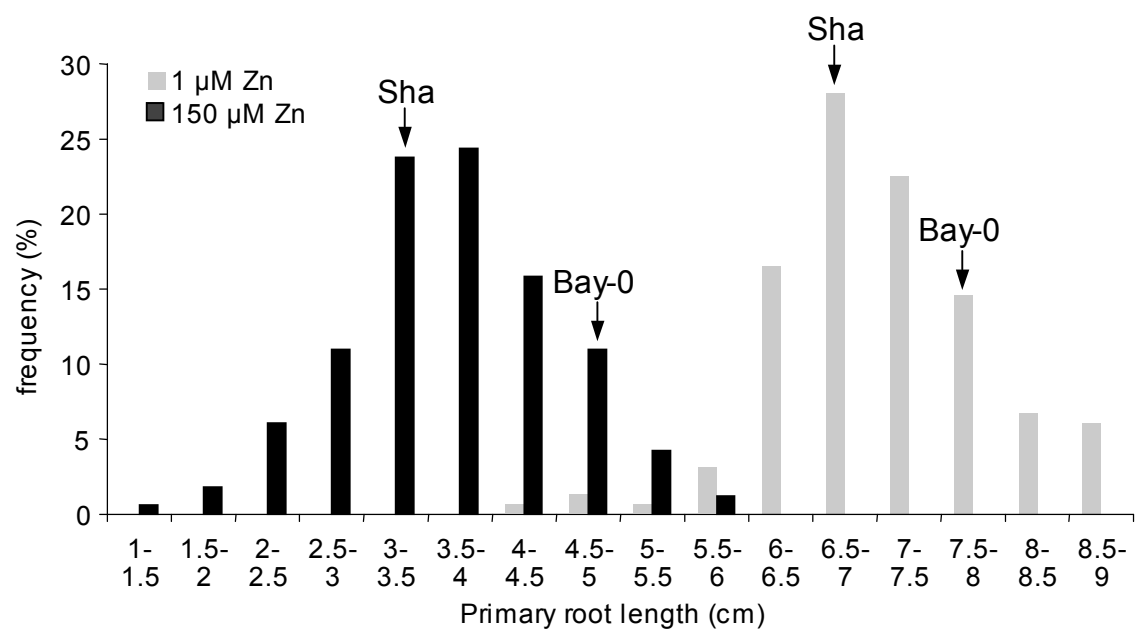

Figure S1

Supplement: Figure S1 — Distribution of the primary root length phenotype among 165 RILs derived from the Bay-0×Shahdara RIL population. Root lengths are from 10-day-old plants grown on agar plates supplemented with 150 µM Zn (black) or not (grey). Mean primary root lengths of the Bay-0 and Shahdara parental lines are indicated. (PDF) [file pgen.1003120.s001.pdf]

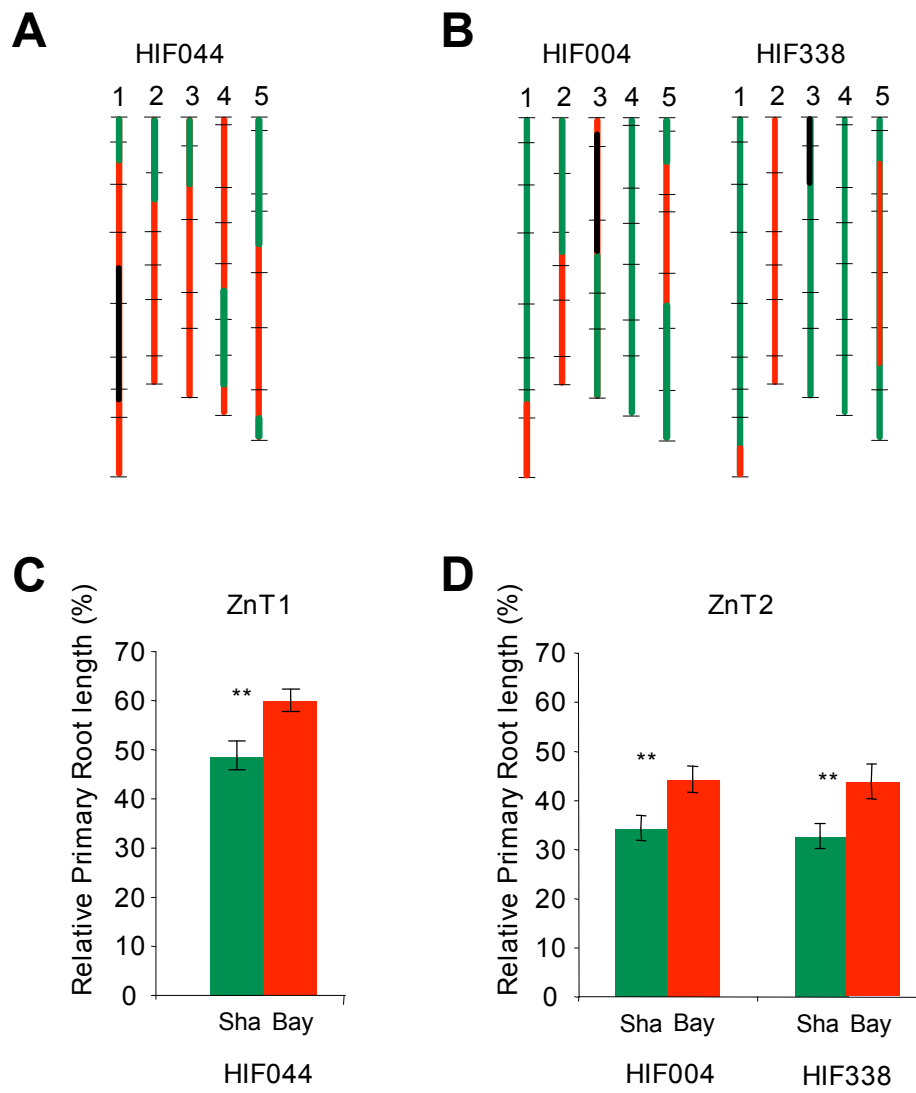

Figure S2

Supplement: Figure S2 — Genotypes of the HIF044 (A), HIF004 and HIF338 (B) lines used to validate ZnT1 (C) and ZnT2 (D) QTLs respectively. HIF044, HIF004 and HIF338 are derived from RIL044, RIL004 and RIL338 that still segregate for the NGA128-MSAT1.13, MSAT302503-MSAT3.19 and NGA172-CAPS7012599 intervals, respectively. (A) (B) Red, green and black portions refer to Bay, Sha and heterozygote genotypes respectively. Horizontal bars represent the positions of the markers used for the genetic mapping of the Bay-0×Shahdara population [4]. The NGA128-MSAT1.13 genomic region includes the ZnT1 support interval and the overlapping MSAT302503-MSAT3.19 and NGA172-CAPS7012599 genomic regions include the ZnT2 support interval. (C) (D) From each of the three RILs, HIF progenies were produced that were fixed at the ZnT loci and thus harbored either the Sha or the Bay allele at these loci. Relative primary root length is the ratio between the primary root length of plants grown at 150 µM and 1 µM Zn respectively and is expressed as a percentage. Error bars represent confidence intervals calculated after a logarithmic transformation of data [41] (** indicates significant differences, P<0.05; n = 14 to 23). (PDF) [file pgen.1003120.s002.pdf]

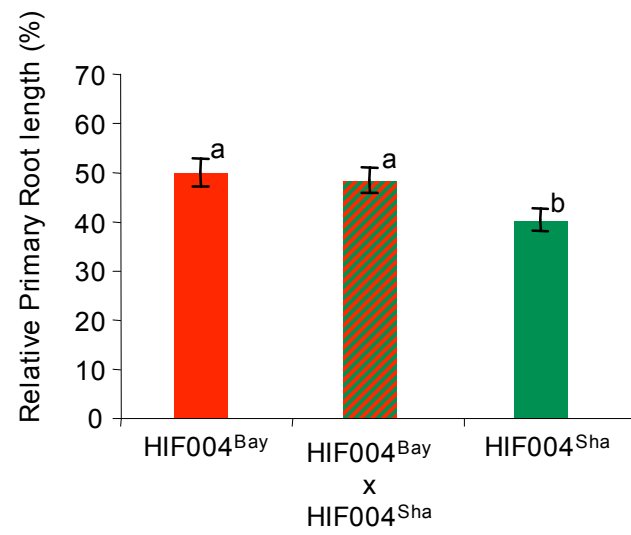

Figure S3

Supplement: Figure S3 — Genetic dominance test at the ZnT2 locus. Relative primary root length is the primary root length of plants grown at 150 µM as a percentage of the primary root length of plants grown at 1 µM Zn. Error bars represent confidence intervals (P<0.05) calculated after a logarithmic transformation of data [41]; n = 9 to 19. Different letters above bars refer to significantly different relative primary root lengths (P<0.05). Reciprocal independent crosses were made between HIF004Bay and HIF004Sha and the Bay allele appeared to be dominant over the Sha allele in phenotypic tests (data not shown). (PDF) [file pgen.1003120.s003.pdf]

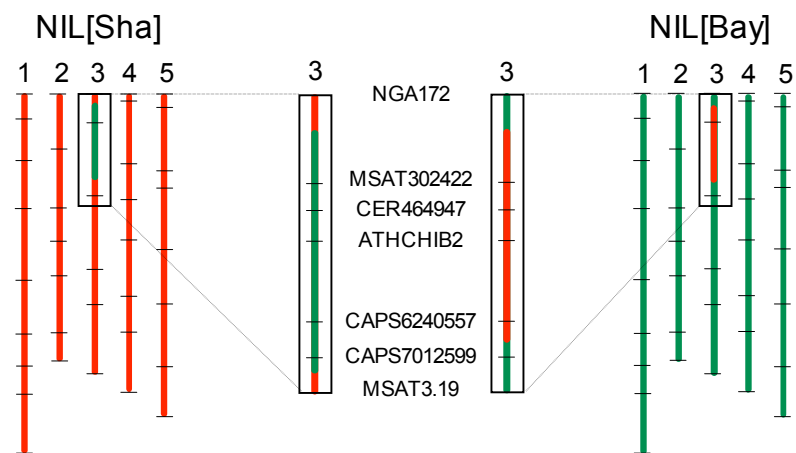

Figure S4

Supplement: Figure S4 — Genotypes of near isogenic lines NIL[Sha] and NIL[Bay]. NIL[Sha] and NIL[Bay] were obtained by back-crossing RIL070 and RIL112 to Bay-0 and Shahdara, respectively. Red and green bars refer to Bay and Sha genotypes respectively. Horizontal bars represent the position of the markers used for the genetic mapping of the RIL population [20]. Newly defined markers are described in Table S3. (PDF) [file pgen.1003120.s004.pdf]

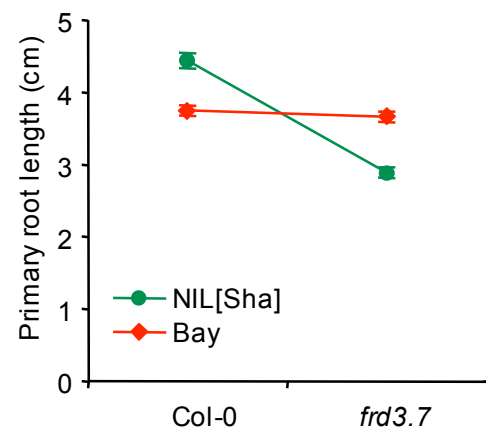

Figure S5

Supplement: Figure S5 — Quantitative complementation test for ZnT2. Primary root length was measured in the presence of 150 µM Zn for F1 plants obtained from crosses of Bay-0 or NIL[Sha] plants with both the Col-0 wild type and frd3.7 mutant plants. F1 plants were genotyped and phenotyped individually. Each value is the mean ± S.E.M., n = 16 to 19. The genotype interaction between the FRD3.7 allele and ZnT2 is highly significant (P<0.001). (PDF) [file pgen.1003120.s005.pdf]

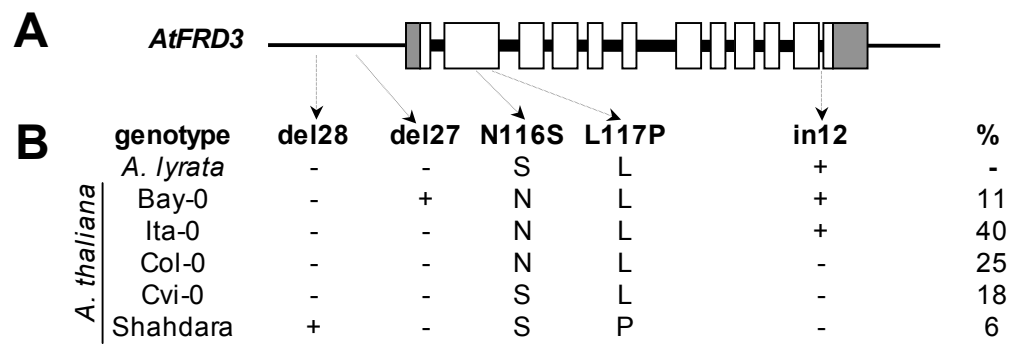

Figure S7

Supplement: Figure S7 — AtFRD3 haplotypes in A. thaliana and A. lyrata. (A) AtFRD3 gene structure. Narrow lines and bold lines represent untranscribed regions and introns respectively. Grey boxes and white boxes refer to untranslated and coding sequences respectively. (B) Five haplotypes were identified using the 5 markers (del28, del27, N116L, S117P, in12) in 109 accessions of A. thaliana. One accession representative of each haplotype is mentioned. The percentage of each haplotype among the 109 accessions is mentioned (%). The 109 accessions are listed in Table S2. The A. lyrata haplotype was deduced from Genbank sequence ADBK01000458. (PDF) [file pgen.1003120.s007.pdf]

**A**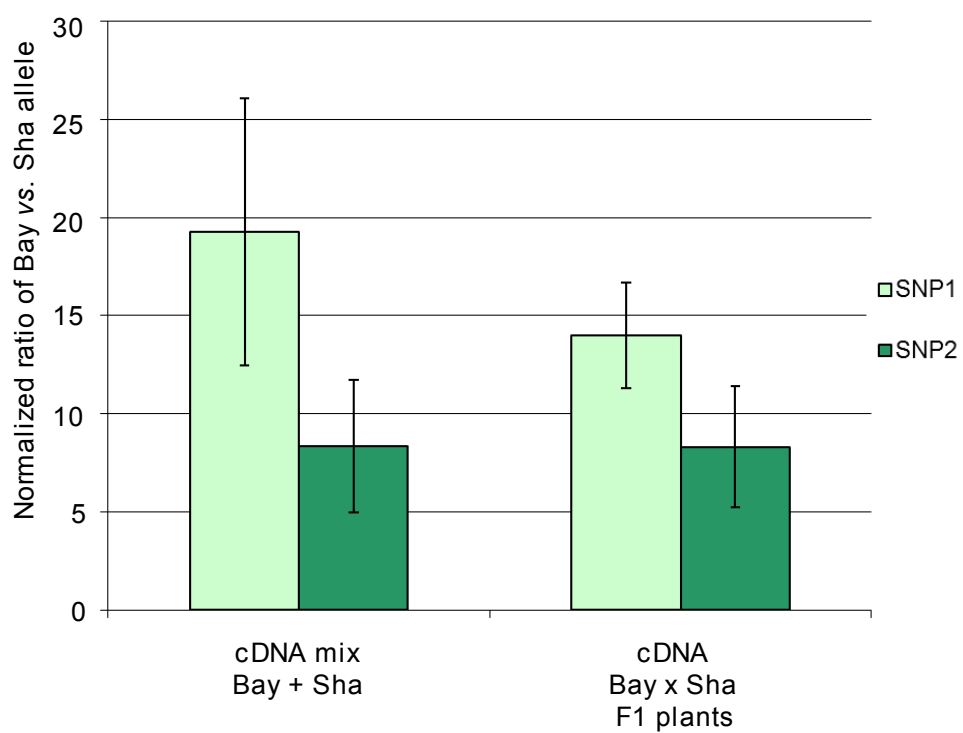**B**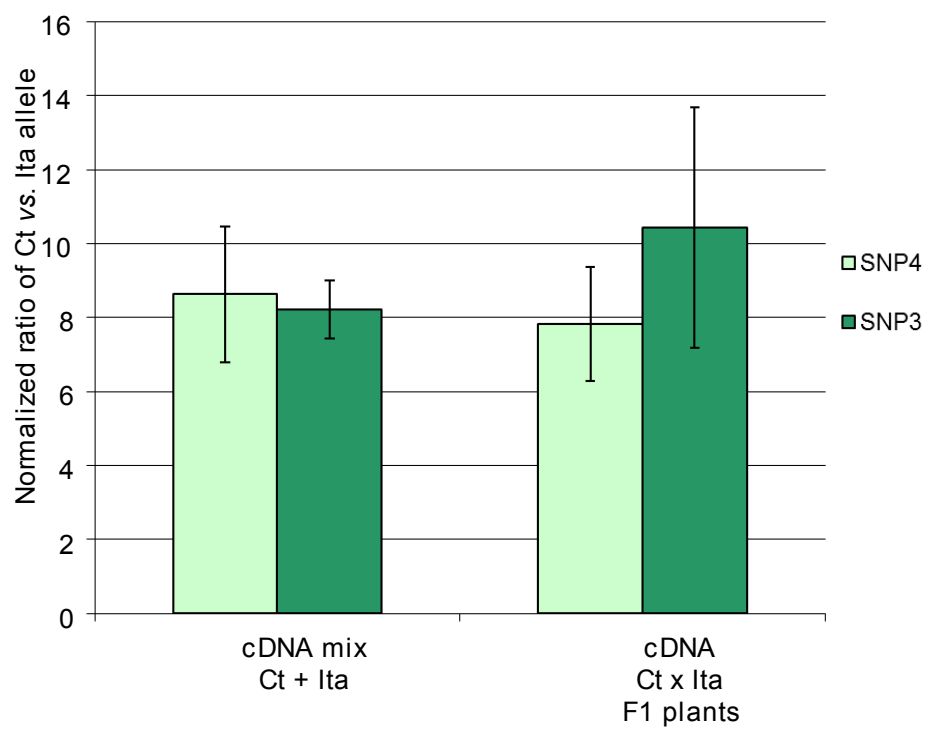

Figure S8

Supplement: Figure S8 — Allele-specific expression assays of FRD3 under Fe shortage. Ratio of allelic expression of Bay-0 vs Shahdara (A) and Ct-1 vs Ita-0 (B) in equal mixtures of parental cDNA and in F1 hybrid cDNA. Values are the mean ± S.E.M. of 6 to 10 ratios from at least three independent pyrosequencing replicates. Two different mRNA SNPs are interrogated per cross. Ratios of allelic expression in F1 hybrid plants are significantly different from 1 (for SNP1, P<4×10^−4^; for SNP2, P<7×10^−3^; for SNP3, P<1×10^−7^; for SNP4, P<1×10^−8^) and not significantly different from those in parental cDNA mixtures, indicating that this differential allelic expression is controlled in cis. (PDF) [file pgen.1003120.s008.pdf]

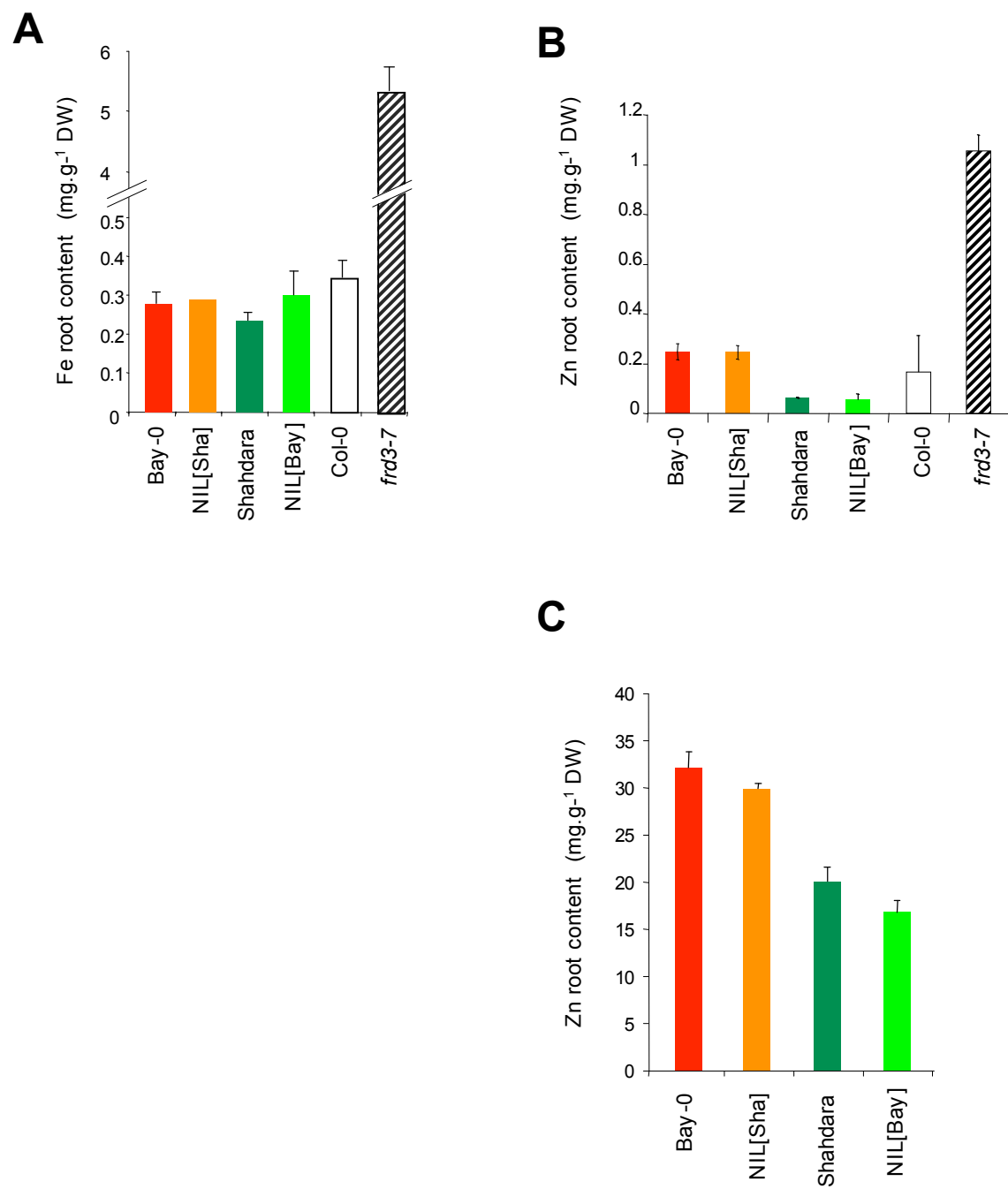

Figure S9

Supplement: Figure S9 — Fe and Zn homeostasis in NILs and parental lines. Fe (A) and Zn (B, C) root contents. Plants were grown on agar plates under control conditions (A, B) or under 200 µM Zn for 10 days before roots were harvested and the Fe and Zn content estimated. Measurements were performed on sets of 5 to 20 plants. Values are the mean ± S.D.M. where n = 4. (PDF) [file pgen.1003120.s009.pdf]

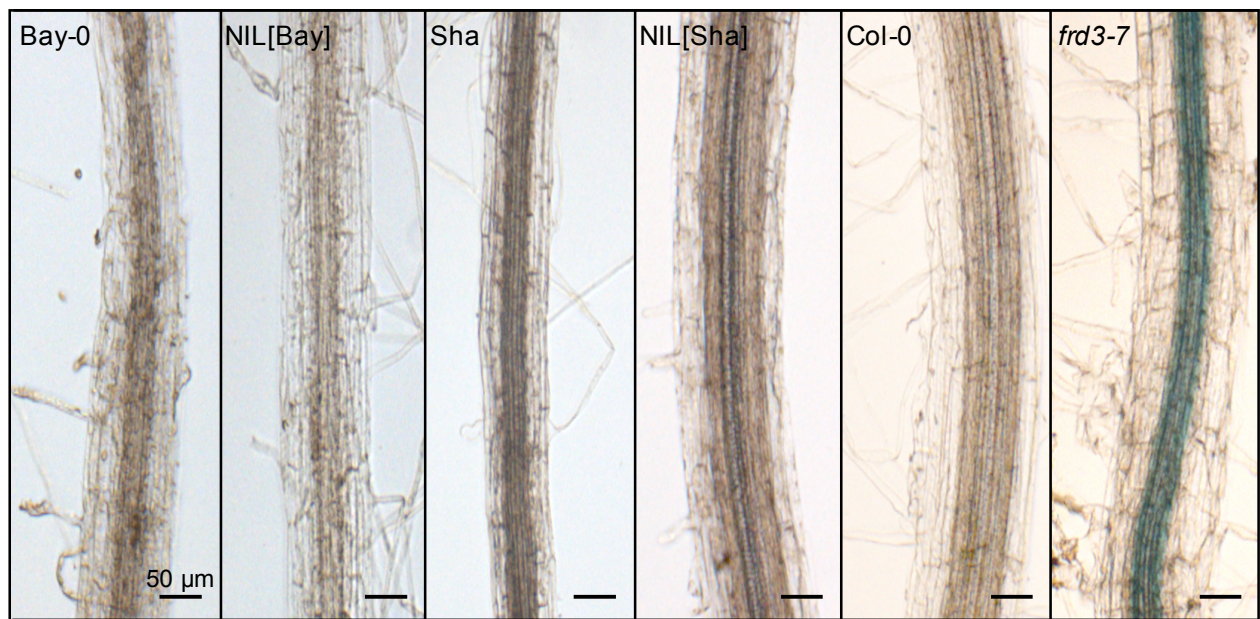

Figure S10

Supplement: Figure S10 — Localization of Fe in roots. Fe accumulation is visualized by Perls staining [21] of intact roots of plants grown on compost for 3 weeks. Only frd3-7 plants accumulate Fe in the xylem. Scale bar, 50 µm. (PDF) [file pgen.1003120.s010.pdf]

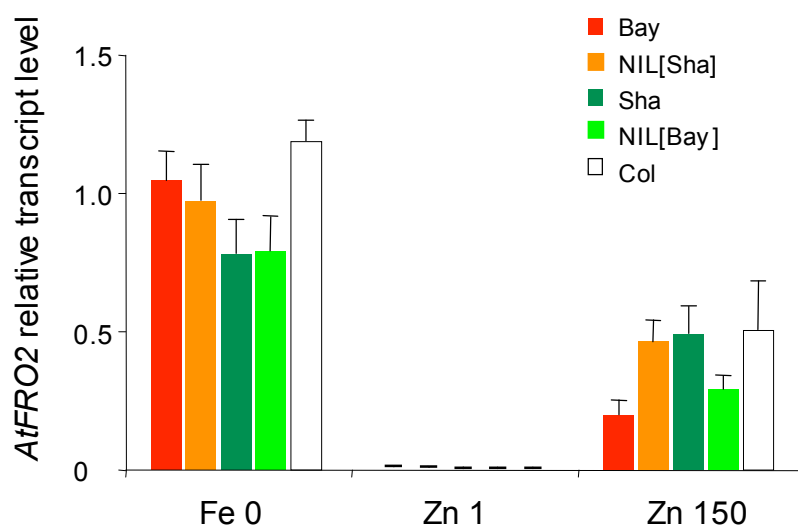

Figure S11

Supplement: Figure S11 — AtFRO2 transcript levels. AtFRO2 (FERRIC REDUCTION OXIDASE 2) is an Fe deficiency marker. Its transcript levels were determined under Fe deficient (Fe0), control (Zn1) and excess Zn (Zn150) conditions in Bay-0 and Shahdara parental lines and in NILs. FRO2 transcript levels are expressed relative to the transcript level of actin (ACT2/ACT8). Values are the means ± S.D.M. where n = 3 independent experiments. (PDF) [file pgen.1003120.s011.pdf]

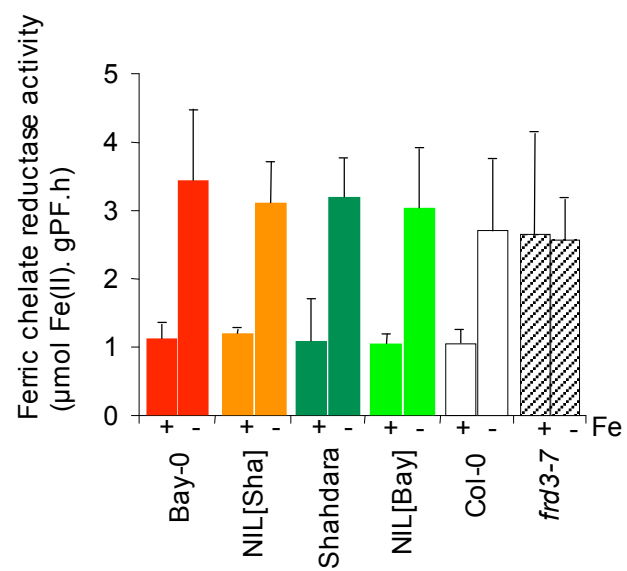

Figure S12

Supplement: Figure S12 — Root ferric chelate reductase activity. Plants were grown on control medium for 7 days and transferred to either iron deficient (−Fe) or iron sufficient (+Fe) medium 4 days before the assay. Values are the mean ± S.D.M. where n = 3 sets of 3 plants. With the exception of frd3-7, all genotypes present a significantly different level of root ferric chelate reductase activity in roots subject to iron deficiency than is found in iron-sufficient treatments (P<0.05, Student test). (PDF) [file pgen.1003120.s012.pdf]

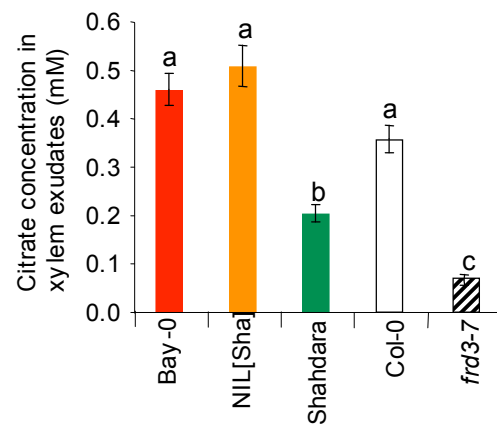

Figure S13

Supplement: Figure S13 — Citrate content of xylem exudates. Plants were grown in a growth chamber for 6 weeks and xylem exudates were collected during 2 h after removing aerial parts of the plant at the hypocotyl. Values are means ± S.E.M. where n = 8 to 12. Different letters above bars refer to significantly different values at P<0.05 according to a nonparametric test. (PDF) [file pgen.1003120.s013.pdf]
