# Supplementary material for: Natural Variation at the FRD3 MATE Transporter Locus Reveals Cross-Talk between Fe Homeostasis and Zn Tolerance in Arabidopsis thaliana
Source: PLoS Genet. 2012 Dec 6;8(12):e1003120. doi: 10.1371/journal.pgen.1003120 (PMC3516540; doi:10.1371/journal.pgen.1003120)
Supplement: Figure S6 — Alignment of the AtFRD3 genomic sequences obtained from the Col-0, Bay-0 and Shahdara accessions of A. thaliana. Annotations refer to the Col-0 sequence. SNPs and indels are shaded. Black letters indicate intergenic and intronic sequences, red letters untranslated regions and green letters coding sequences. Start and stop codons are indicated in bold letters. (PDF) [file pgen.1003120.s006.pdf]

```
FRD3_COL  TGACGTTGATGGCTTGAAAAAGATGGCCTTATCTTGCGGAACAAACAATT
FRD3_BAY  TGACGTTGATGGCTTGAAAAAGATGGCCTTATCTTGCGGAACAAACAATT
FRD3_SHA  TGACGTTGATGGCTTGAAAAAGATGGCCTTATCTTGCGGAACAAACAATT

FRD3_COL  ACATACACGACACGCACTATATACAACTCACCTGTGTTGGTCTCTGTTGC
FRD3_BAY  ACATACACGACACGCACTATATACAACTCACCTGTGTTGGTCTCTGTTGC
FRD3_SHA  ACATACACGACACGCACGATATACAACTCACCTGTGTTGGTCTCTGTTGC

FRD3_COL  CATCTTTTATGTTATTGTTTTCCGACTGTCGCCTTCCTTTAACTAATATT
FRD3_BAY  CATCTTTTATGTTATTGTTTTCCGACTGTCGCCTTCCTTTAACTAATATT
FRD3_SHA  CATCTTTTATGTTATTGTTTTCCGACTGTCGCCTTCCTTTAACTAATATT

FRD3_COL  ATAATTTTAAAGATGTTCATAAATCACAGTAGAAAGCTTGTTTTTGCTAA
FRD3_BAY  ATAATTTTAAAGATGTTCATAAATCACAGTAGAAAGCTTGTTTTTGCTAA
FRD3_SHA  ATAATTTTAAAGATGTTCATAAATCACAGTAGAAAGCTTGTTTTTGCTAA

FRD3_COL  AATGAACATGACACGGATCATACAAAAAATATATTTTACACTATAGCTAT
FRD3_BAY  AATGAACATGACACGGATCATACAAAAAATATATTTTACACTATAGCTAT
FRD3_SHA  AATGAACATGACACGGATCATACAAAAAATATATTTTACACTATAGCTAT

FRD3_COL  ATACCGATTTAATCTTAGGTACTTTGAATCGTGCTAAAACTAAACGGCCT
FRD3_BAY  ACACCGATTTAATCTTAGGTACTTTGAATCGTGCTAAAACTAAACGGCCT
FRD3_SHA  ATACCGATTTAATCTTAGGTACTTTGAATCGTGCTAAAACTAAACGGCCT

FRD3_COL  TCTCAAAACCCTCCCTCTTTTCCTCCCTCCCTCAGAAACCCCTCCATCGA
FRD3_BAY  TCTCAAAACCCTCCCTCTTTTCCTCCCTCCCTCAGAAACCCCTCCATCGA
FRD3_SHA  TCTCAAAACCCTCCCTCTTTTCCTCCCTCCCGCAGAAACCCCTCCCTCGA

FRD3_COL  CAAATAACGTTATGCAATTCTCTAAACAATGCTCGACAAGCATGTGTTTT
FRD3_BAY  CAAATAACGTTATGCAATTCTCTAAACAATGCTCGACAAGCATGTGTTTT
FRD3_SHA  CAAATAACGTTATGCAATTCTCTAAACAATGCTCGACAAGCATGTGTTTT

FRD3_COL  TAGTAATGCTACAACTTATTTCTCTTTTCAACGTCCTAAGAGGCATCAAA
FRD3_BAY  TAGTAATGCTACAACTTATTTCTCTTTTCAACGTCCTAAGAGGCATCAAA
FRD3_SHA  TAGTAATGCTACAACTTATTTCTCTTTTCAACGTCCTAAGAGGCATCAAA

FRD3_COL  AAGATCAAAGATCTTGGAACCGAGGTCCTATGCAAGGAATCATTATAAAG
FRD3_BAY  AAGATCAAAGATCTTGGAACCGAGGTCCTATGCAAGGAATCATTATAAAG
FRD3_SHA  AAGATCAAAGATCTTGGAACCGAGGTCCTATGCAAGGAATCATTATAAAG

FRD3_COL  TACCATGTTATTTTTTTAAATAACATCGTTTTCTTAATATAATTTATAAA
FRD3_BAY  TACCATGTTATTTTTTTAAATAACATCGTTTTCTTAATATAATTTATAAA
FRD3_SHA  TACCATGTTATTTTTTTAAATAACATTGTTTTCTTAATATAATTTACAAA

FRD3_COL  TACCGTTATTTTTACCGAAATTTCATATATATGTCAGTTTTATACTTTGT
FRD3_BAY  TACCGTTATTTTTACCGAAATTTCATATATATGTCAGTTTTATACTTTGT
FRD3_SHA  TACCGTTATTTTTACCGAAATTTCATATCTATGTCAGTTTTATACTTTGT

FRD3_COL  ACGATAACGCCAAAAACTTTAATTATCGCCAAAATTGTAAACGGTATTTT
FRD3_BAY  ACGATAACGCCAAAAACTTTAATTATCGCCAAAATTGTAAACGGTATTTT
FRD3_SHA  ACGATAACGCCAAAAACTTTAATTATCGCCAAAATTGTAAACGGTATTTT

FRD3_COL  CGTCGTTTAGTTATTTACCAAAAATAAAATGACGATTGCAACTTATTTAG
FRD3_BAY  CGTCGTTTAGTTATTTACCAAAAATAAAATGACGATTGCAACTTATTTAG
FRD3_SHA  CGTCGTTTAGTTATTTACCAAAAATAAAATGACGATTGCAACTTCTTTAG

FRD3_COL  TTAAAATACAAAAAAAAACTAATATATTAATTGAGCGGACGGAATTTTTT
FRD3_BAY  TTAAAATACAAAAAAAA-CTAATATATTAATTGAGCGGACGGAATTTTTT
FRD3_SHA  TTAAAATACAAAAAAA--CTAATATATTAATTGAGCGGACGAAATTTTTT

FRD3_COL  TCCAAAATCCCGATGTGTAAATATGAGAACGTTTCGAGGATAACTTACAA
FRD3_BAY  TCCAAAATCCCGATGTGTAAATATGAGAACGTTTCGAGGATAACTTACAA
FRD3_SHA  TCCAAAATCCCGATGTGTAAATATGAGAACGTTTCGAGGATAACTTACAA

FRD3_COL  ATTAAACATTAATAAAAATGATAAAGTGTAGTTAGGAGCTAAATTGTGAT
FRD3_BAY  ATTAAACATTAATAAAAATGATAAAGTGTAGTTAGGAGCTAAATTGTGAT
FRD3_SHA  ATTAAACATTAATCAAAATGATAAAGTGTAGTTAGGAGCTAAATTGTGAT

FRD3_COL  AGTAAACATCTATCTCTAATATTATTAAATGAATTATAATACTATTTTAA
FRD3_BAY  AGTAAACATCTATCTCTAATATTATTAAATGAATTATAATACTATTTTAA
FRD3_SHA  AGTAAACATCTATCTCTAATATTATTAAATGAATTATAATAATATTTTAA

FRD3_COL  TCATAGTATTAAATTTCTTTAATTAAAAATATAAATAATTTCAATTTAAT
FRD3_BAY  TCATAGTATTAAATTTCTTTAATTAAAAATATAAATAATTTCAATTTAAT
FRD3_SHA  TTATAGTATCAAATTTCTTTAATTAAAAATATAAATAATTTCAATTTAAT

FRD3_COL  TCTATACCAAATTAACCCGAAAATATTTTATCTAACATACACAAAGACAC
FRD3_BAY  TCTATACCAAATTAACCCGAAAATATTTTATCTAACATACACAAGGACAC
FRD3_SHA  TCTATACCAAATTAACCCAAAAATATTCTATCTAACATA-ACAAAGACAC

FRD3_COL  ATAAAAGTTTTGATAACTGCCTAAAAAAAATAAGCTTTTGAATTATTAATT
FRD3_BAY  ATAAAAGTTTTGATAACTGCCTAAAAAAAATAAGCTTTTGAATTATTAATT
FRD3_SHA  ATAAAAGTTTTGATAACTGCCTAAAAAAAATAAGCTTTTAAATTATTAATT

FRD3_COL  AGTTGTTATTCAATGATAAAATAACATTATTTGTCAACTAGTGAATTCCA
FRD3_BAY  AGTTGTTATTCAATGATAAAATAACATTATTTGTCAACTAGTGAATTCCA
FRD3_SHA  AGATAT----------------------------CAACTAGTGAATTTCA

FRD3_COL  ATTACGCAAAATGATTCACTTTTTTAGTGGAAAATATCAAAGAAAAATGA
FRD3_BAY  ATTACGCAAAATGATTCACTTTTTTAGTGGAAAATATCAAAGAAAAATGA
FRD3_SHA  ATTACGCAAAATGATTCACTTTTTTAGTGGAAAATATCAAAGAAAAATGA

FRD3_COL  GAAGTTTATATGAAAATAAACTCTTTCCCACTATGATGAATACATGTAAG
FRD3_BAY  GAAGTTTATATGAAAATAAACTCTTTCCCACTATGATGAATACATGTAAG
FRD3_SHA  GAAGTTTATATGAAAATAAACTCTTTCCCACTATGATGAATACATGTAAG

FRD3_COL  AAAACTTTCATGAAAAGAAAACTTATTTACTCAATATAAAAATAGAAGAC
FRD3_BAY  AAAACTTTCATGAAAAGAAAACTTATTTACTCAATATAAAAATAGAAGAC
FRD3_SHA  AAAACTTTCATGAAAAGAAAACTTATTTACTCAATATAAAAATAGAAGAC

FRD3_COL  TCTTTATCTTTCACGAGTAAAAGTTCACGAAAACCATATTTTCCTATTGA
FRD3_BAY  TCTTTCTCTTTCACGAGTAAAAGTTCACGAAAACCATATAATCCTATTGA
FRD3_SHA  TCTTTATCTTTCACGAGTAAAAGTTCACGAAAACCATATTTTCCTATTGA

FRD3_COL  TTAAAGAAATCATAGAAGTTAAAATAATCAACAAGGGCAAGCCAAAAACT
FRD3_BAY  TTAA---------------------------CAAGGGCAAGCCGAAAACT
FRD3_SHA  TTAAAGAAATCATAGAAGTTAAAATAATCAACAAGGGCAAGCCAAAAACT

FRD3_COL  TCTAGTGTGGGATTTACTTAATAGAAGTATATATATTACGATGTTTATGC
FRD3_BAY  TCTAGTG--GGATTTACTTAATAGAAGTATATATATTACGATGTTTATGC
FRD3_SHA  TCTAGTGTGGGATTTACTTAATAGAAGTATATATATTACGATGTTTATGC

FRD3_COL  GTACCTATTTTCCCTCAATGAGAAGAGAAATTCCATAATATTGGTGTCTT
FRD3_BAY  GTACCTATTTTCCCTCAATGAGAAGAGAAATTCCATAATATTGGTGTCTT
FRD3_SHA  GTACCTATTTTCCCTCAATGAGAAGAGAAATTCCATAATATTGGTGTCTT

FRD3_COL  AAGTTTGGACGGAAATAAAGAGCAGCAAAAAAGTTAGGGAAGGAAACCTT
FRD3_BAY  AAGTTTGGACGGAAATAAAGAGCAGCAAAAAAGTTAGGGAAGGAAACCTT
FRD3_SHA  AAGTTTGGACGGAAATAAAGAGCAGCAAAAAAGTTAGGGAAGGAAACCTT

FRD3_COL  TGTTTTCTTCAATAATTATAGAAAATAATTTCTTTTATTG--ATTTAGAT
FRD3_BAY  TGTTTTCTTCAATAATTATAGAAAATAATTTAATTTATTTTTATTTAGAT
FRD3_SHA  TGTTTTCTTCAATAATTATAGAAAATAATTTCTTTTATTG--ATTTAGAT

FRD3_COL  ATTAAATAAGCAAAGATATGCATGCTCATTACGTGTCTATAAATAAAAAC
FRD3_BAY  ATTAAATAAGCAAAGATATGCATGCTCATTACGTGTCTATAAATAAAAAC
FRD3_SHA  ATTAAATAAGCAAAGATATGCATGCTCATTACGTGTCTATAAATAAAAAC

FRD3_COL  ACGTTTGTACATAGCATCTACTATAAACGTTCCTTTTGCTTCCCCGATTC
FRD3_BAY  ACGTTTGTACATAGCATCTACTATAAACGTTCCTTTTGCTTCCCCGATTC
FRD3_SHA  ACGTTTGTACATAGCATCTACTATAAACGTTCCTTTTGCTTCCCCGATTC

FRD3_COL  TTCGAAACACTTATTGATATCTTCAGACACAACAAATTAATTACAGAGAC
FRD3_BAY  TTCGAAACACTTATTGATATCTTCAGACACAACAAATTAATTACAGAGAC
FRD3_SHA  TTCGAAACACTTATTGATATCTTCAGACACAACAAATTAATTACAGAGAC

FRD3_COL  AGTTACAGAGGAAAAAGATCTATGACGGAAACTGGTGATGATCTTGCTAC
FRD3_BAY  AGTTACAGAGGAAAAAGATCTATGACGGAAACTGGTGATGATCTTGCTAC
FRD3_SHA  AGTTACAGAGGAAAAAGATCTATGACGGAAACTGGTGATGATCTTGCTAC

FRD3_COL  GGTGAAGAAGCCAATCCCATTTCTCGTTATCTTCAAAGATTTAAGGTGTG
FRD3_BAY  GGTGAAGAAGCCAATCCCATTTCTCGTTATCTTCAAAGATTTAAGGTGTG
FRD3_SHA  GGTGAAGAAGCCAATCCCATTTCTCGTTATCTTCAAAGATTTAAGGTGTG

FRD3_COL  TGTTTATGTATTCATGAAATGGTGATGAAATTTTTGAAAGAAGTGATGCA
FRD3_BAY  TGTTTATGTATTCATGAAATGGTGATGAAATTTTTGAAAGAAGTGATGCA
FRD3_SHA  TGTTTATGTATTCATGAAATGGTGATGAAATTTTTGAAAGAAGTGATGCA

FRD3_COL  TAACATTAGTTTATTTATGTAAAATTGCAGACATGTATTCAGTAGGGACA
FRD3_BAY  TAACATTAGTTTATTTATGTAAAATTGCAGACATGTATTCAGTAGGGACA
FRD3_SHA  TAACATTAGTTTATTTATGTAAAATTGCAGACATGTATTCAGTAGGGACA

FRD3_COL  CAACTGGGCGAGAGATTCTAGGCATCGCGTTTCCAGCAGCTTTGGCTTTA
FRD3_BAY  CAACTGGGCGAGAGATTCTAGGCATCGCGTTTCCAGCAGCTTTGGCTTTA
FRD3_SHA  CAACTGGGCGAGAGATTCTAGGCATCGCGTTTCCAGCAGCTTTGGCTTTA

FRD3_COL  GCTGCTGATCCAATCGCTTCTCTGATTGATACCGCTTTTGTCGGGCGTTT
FRD3_BAY  GCTGCTGATCCAATCGCTTCTCTGATTGATACCGCTTTTGTCGGGCGTTT
FRD3_SHA  GCTGCTGATCCAATCGCTTCTCTGATTGATACCGCTTTTGTCGGGCGTTT

FRD3_COL  AGGAGCGGTTCAGCTAGCGGCGGTTGGAGTTTCCATTGCCATATTCAATC
FRD3_BAY  AGGAGCGGTTCAGCTAGCGGCGGTTGGAGTTTCCATTGCCATATTCAATC
FRD3_SHA  AGGAGCGGTTCAGCTAGCGGCGGTTGGAGTTTCCATTGCCATATTCAATC

FRD3_COL  AAGCTTCTAGAATTACGATATTCCCACTTGTGAGCCTCACAACTTCATTT
FRD3_BAY  AAGCTTCTAGAATTACGATATTCCCACTTGTGAGCCTCACAACTTCATTT
FRD3_SHA  AAGCTTCTAGAATTACGATATTCCCACTTGTGAGCCTCACAACTTCATTT

FRD3_COL  GTGGCAGAGGAAGACACGATGGAGAAGATGAAAGAAGAAGCAAACAAAGC
FRD3_BAY  GTGGCAGAGGAAGACACGATGGAGAAGATGAAAGAAGAAGCAAACAAAGC
FRD3_SHA  GTGGCAGAGGAAGACACGATGGAGAAGATGAAAGAAGAAGCAAACAAAGC

FRD3_COL  CAATCTTGTTCATGCAGAAACTATACTTGTTCAAGATTCTTTGGAAAAGG
FRD3_BAY  CAATCTTGTTCATGCAGAAACTATACTTGTTCAAGATTCTTTGGAAAAGG
FRD3_SHA  CAGTCCTGTTCATGCAGAAACTATACTTGTTCAAGATTCTTTGGAAAAAG

FRD3_COL  GCATTTCTTCACCTACAAGTAACGATACCAACCAGCCACAGCAACCTCCA
FRD3_BAY  GCATTTCTTCACCTACAAGTAATGATACCAACCAGCCACAGCAACCTCCA
FRD3_SHA  GCATTTCTTCACCTACAAGTAATGATACCAACCAGCCACAGCAACCTCCA

FRD3_COL  GGTAAATTCCGCATATCTCACTCGACATTGATAACTTTTATTAAAGTTTC
FRD3_BAY  GGTAAATTCCGCATATCTCACTCGACATTGATAACTTTTATTAAAGTTTC
FRD3_SHA  GGTAAATTCCGCATATCTCACTCGACATTGATAACTTTTATTAAAGTTTC

FRD3_COL  GATTGTTTTTTTACTGTTGGTTTCTTCTCTCGATCTCTTTTGTTTCAATT
FRD3_BAY  GATTGTTTTTTTACTGTTGGTTTCTTCTCTCGATCTCTTTTGTTTCAATT
FRD3_SHA  GATTGTTTTTTTACTGTTGGTTTCTTCTCTCGATCTCTTTTGTTTCAATT

FRD3_COL  TGTTGTTTTTTTTGGTTGTATTAAACTTAGCTCCGGATACAAAGTCAAATA
FRD3_BAY  TGTTGTTTTTTTTGGTTGTATTAAACTTAGCTCCGGATACAAAGTCAAATA
FRD3_SHA  TGTTGTTTTTTTTGGTTGTATTAAACTTAGCTCCGGATACAAAGTCAAATA

FRD3_COL  GCGGAAACAAATCGAATAAAAAGGAGAAGAGGACCATTAGAACAGCATCA
FRD3_BAY  GCGGAAACAAATCGAATAAAAAGGAGAAGAGGACCATTAGAACAGCATCA
FRD3_SHA  GCGGAAACAAATCGAATAAAAAGGAGAAGAGGACCATTAGAACAGCATCA

FRD3_COL  ACAGCTATGATCTTGGGGTTAATCCTTGGCCTTGTGCAAGCTATTTTCTT
FRD3_BAY  ACAGCTATGATCTTGGGGTTAATCCTTGGCCTTGTGCAAGCTATTTTCTT
FRD3_SHA  ACAGCTATGATCTTGGGGTTAATCCTTGGCCTTGTGCAAGCTATTTTCTT

FRD3_COL  GATTTTCAGTTCAAAGTTGCTTCTAGGCGTCATGGGAGTGAAACCAGTAA
FRD3_BAY  GATTTTCAGTTCAAAGTTGCTTCTAGGCGTCATGGGAGTGAAACCAGTAA
FRD3_SHA  GATTTTCAGTTCAAAGTTGCTTCTAGGCGTCATGGGAGTGAAACCAGTAA

FRD3_COL  GTTTTCAGAAATATACATATTTTGTTGGGATCTATAGCATAAAATGTTTT
FRD3_BAY  GTTTTCAGAAATATACATATTTTGTTGGGATCTATAGCATAAAATGTTTT
FRD3_SHA  GTTTTCAGAAATATACATATTTTGTTGGGATCTATAGCATAAAATGTTTT

FRD3_COL  GACTAATTTGAGTTGAATTTGGATAACAGAATTCACCAATGTTATCACCA
FRD3_BAY  GACTAATTTGAGTTGAATTTGGATAACAGAATTCACCAATGTTATCACCA
FRD3_SHA  GACTAATTTGAGGTGAATTTGGATAACAGAATTCACCAATGTTATCACCA

FRD3_COL  GCACACAAGTACTTGAGCATACGAGCTTTGGGGGCTCCTGCATTGCTTCT
FRD3_BAY  GCACACAAGTACTTGAGCATACGAGCTTTGGGGGCTCCTGCATTGCTTCT
FRD3_SHA  GCACACAAGTACTTGAGCATACGAGCTTTGGGGGCTCCTGCATTGCTTCT
```

Figure S6

```
FRD3_COL ATCTCTTGCTATGCAAGGCATCTTTCGTGGATTCAAGGACACCAAAACTC
FRD3_BAY ATCTCTTGCTATGCAAGGCATCTTTCGTGGATTCAAGGACACCAAAACTC
FRD3_SHA ATCTCTTGCTATGCAAGGCATCTTTCGTGGATTCAAGGACACCAAAACTC

FRD3_COL CTCTCTTTGCCACTGGTAATTAAGTTGTTAACTTAGATCATCTTTAATGA
FRD3_BAY CTCTCTTTGCCACTGGTAATTAAGTTGTTAACTTAGATCATCTTTAATGA
FRD3_SHA CTCTCTTTGCCACTGGTAATTAAGTTGTTAAATTAGATCATCTTTAATGA

FRD3_COL TCACTCTCCTTACTTCTTATAATAT-----------------TTTGCCTT
FRD3_BAY TCACTCTCCTTACTTCTTATAATATGTTAGCTAATATAATATTTTGCCTT
FRD3_SHA TCACTTTTCTTACTTCTTATAATATGTTAGCTAATATAATATCTTGCCTT

FRD3_COL AATGCGTGAAACAGTCGTAGCAGATGTTATCAACATAGTTCTCGACCCCA
FRD3_BAY AATGCGTGAAACAGTCGTAGCAGATGTTATCAACATAGTTCTCGACCCCA
FRD3_SHA AATGCGTGAAACAGTCGTAGCAGATGTTATCAACATAGTTCTCGACCCCA

FRD3_COL TCTTCATTTTTGTGCTTCGTCTAGGGATCATCGGTGCAGCCATTGCCCAT
FRD3_BAY TCTTCATTTTTGTGCTTCGTCTAGGGATCATCGGTGCAGCCATTGCCCAT
FRD3_SHA TCTTCATTTTTGTGCTTCGTCTAGGGATCATCGGTGCAGCCATTGCCCAT

FRD3_COL GTCATTTCTCAGTAAGAGAAATCACTAAAAAAAATTCCACACATGCAAAAG
FRD3_BAY GTCATTTCTCAGTAAGAGAAATCACTAAAAAAAATTCCACACATGCAAAAG
FRD3_SHA GTCATTTCTCAGTAAGAGAAATCACTAAAAAAAATTCCACACATGCAAAAG

FRD3_COL TGATCATTATTGAACAAAATCGCTAGGCGCACTCTTGTTTTTCTACAGCT
FRD3_BAY TGATCATTATTGAACAAAATCGCTAGGCGCACTCTTGTTTTTCTACAGCT
FRD3_SHA TGATCATTATTGCACAAAATCGCTAGGCGCACTCTTGTTTTTCTACAGCT

FRD3_COL ATAAATAGACTTGTGAAGTCATAACCTCAAACAAAAACAAATGATTTGTT
FRD3_BAY ATAAATAGACTTGTGAAGTCATAACCTCAAACAAAAACAAATGATTTGTT
FRD3_SHA ATAAATAGACTTGTGAAGTCATAACCTCAAACAAAAACTAATGATTTGTT

FRD3_COL TGTGTACGTGAAGGTACTTCATGACTCTAATATTGTTCGTCTTCCTCGCA
FRD3_BAY TGTGTACGTGAAGGTACTTCATGACTCTAATATTGTTCGTCTTCCTCGCA
FRD3_SHA TGTGTACGTGAAGGTACTTCATGACTCTAATATTGTTCGTCTTCCTCGCA

FRD3_COL AAGAAAGTTAATTTGATTCCACCAAACTTCGGGGATTTGCAGTTTGGAAG
FRD3_BAY AAGAAAGTTAATTTGATTCCACCAAACTTCGGGGATTTGCAGTTTGGAAG
FRD3_SHA AAGAAAGTTAATTTGATTCCACCAAACTTCGGGGATTTGCAGTTTGGAAG

FRD3_COL GTTCCTTAAAAATGGTACGTATGGATGCATATTTATTAAAAGTTGTGGTT
FRD3_BAY GTTCCTTAAAAATGGTACGTATGGATGCATATTTATTAAAAGTTGTGGTT
FRD3_SHA GTTCCTTAAAAATGGTACGTATGGATGCATATTTATTAAAAGTTGTGGTT

FRD3_COL CTTGCAATAATATTTTTTTTT-AAAAACAAGATCCGTCGTAGGAGCTAAT
FRD3_BAY CTTGCAATAATATTTTTTTTTTAAAAACAAGATCCGTCGTAGGAGCTAAT
FRD3_SHA CTTGCAATAATAATTTTTTTT-AAAAACAAGATCCGTCGTAGGAGCTAAT

FRD3_COL GCACAGAGTCCAAAAATAAATTAACAAAAAATTTATCTATATAATAATAG
FRD3_BAY GCACAGAGTCCAAAAATAAATTAACAAAAAATTTATCTATATAATAATAG
FRD3_SHA GCACAGAGTCCAAAAATAAATTAACAAAAAATTTATCTATATAATAATAG

FRD3_COL AATTCAATCAAATAAGGTCTATATTTAAAATATTGAATATTTTGAAATAT
FRD3_BAY AATTCAATCAAATAAGGTCTATATTTAAAATATTGAATATTTTGAAATAT
FRD3_SHA AATTCAATCAAATAAGGTCTATATTTAAAATATTGAATATTTTGAAATAT

FRD3_COL ATAGTTAAGAAAATGAGAAATGTGGATATATGTCTAACAAGTATAGTATT
FRD3_BAY ATAGTTAAGAAAATGAGAAATGTGGATATATGTCTAACAAGTATAGTATT
FRD3_SHA ATAGTTAAGAAAATGAGAAATGTGGATATATGTCTAACAAGTATAGTATT

FRD3_COL AAAAATGAAAGGGCTACTATTGCTGGCGAGGACCATAGCAGTGACGTTTT
FRD3_BAY AAAAATGAAAGGGCTACTATTGCTGGCGAGGACCATAGCAGTGACGTTTT
FRD3_SHA AAAAATGAAAGGGCTACTATTGCTGGCGAGGACCATAGCAGTGACGTTTT

FRD3_COL GTCAGACCTTAGCAGCAGCAATGGCGGCGCGGCTGGGTACAACACCAATG
FRD3_BAY GTCAGACCTTAGCAGCAGCAATGGCGGCGCGGCTGGGTACAACACCAATG
FRD3_SHA GTCAGACCTTAGCAGCAGCAATGGCGGCGCGGCTGGGTACAACACCAATG

FRD3_COL GCTGCTTTTCAGATTTGTTTACAAGTATGGTTAACTTCTTCTCTTCTCAA
FRD3_BAY GCTGCTTTTCAGATTTGTTTACAAGTATGGTTAACTTCTTCTCTTCTCAA
FRD3_SHA GCTGCTTTTCAGATTTGTTTACAAGTATGGTTAACTTCTTCTCTTCTCAA

FRD3_COL TGATGGTCTTGCCGTTGCTGGTCAGGTAATCATGTTTTCTCGTTGTATTA
FRD3_BAY TGATGGTCTTGCCGTTGCTGGTCAGGTAATCATGTTTTCTCGTTGTATTA
FRD3_SHA TGATGGTCTTGCCGTTGCTGGTCAGGTAATCATGTTTTCTCGTTGTATTA

FRD3_COL ATTTATGTATAGTTTATATGGTTGATCAAGTTGTATGTAGAAAATGATCA
FRD3_BAY ATATATGTATAGTTTATATGGTTGATCAAGTTGTATGTAGAAAATGATCA
FRD3_SHA ATATATGTATAGTTTATATGGTTGATCAAGTTGTATGTAGAAAATGATCA

FRD3_COL TTCAATACGTTGCAGGCGATTCTGGCTTGTTCGTTTGCTGAGAAGGACTA
FRD3_BAY TTCAATACGTTGCAGGCGATTCTGGCTTGTTCGTTTGCTGAGAAGGACTA
FRD3_SHA TTCAATACGTTGCAGGCGATTCTGGCTTGTTCGTTTGCTGAGAAGGACTA

FRD3_COL TAACAAAGTGACTGCTGTTGCATCCCGTGTTCTACAGGTTCGGTCCAAAA
FRD3_BAY TAACAAAGTGACTGCTGTTGCATCCCGTGTTCTACAGGTTCGGTCCAAAA
FRD3_SHA TAACAAAGTGACTGCTGTTGCATCCCGTGTTCTACAGGTTCGGTCCAAAA

FRD3_COL ATCACATTACCAAACCTTTCTTTAAAAATAAAATAATTGTGTAACTAAAA
FRD3_BAY ATCACATTACCAAACCTTTCTTTAAAAATAAAATAATTGTGAAACTAAAA
FRD3_SHA ATCACATTACCAAACCTTTCTTTAAAAATAAAATAATTGTGTAACTAAAA

FRD3_COL CAGAAATGAATTTGATACGCAGATGGGTTTTGTGTTAGGACTTGGACTGT
FRD3_BAY CAGAAATGAATTTGATACGCAGATGGGTTTTGTGTTAGGACTTGGACTGT
FRD3_SHA CAGAAATGAATTTGATACGCAGATGGGTTTTGTGTTAGGACTTGGACTGT

FRD3_COL CCGTTTTTGTTGGACTAGGTCTCTACTTTGGTGCCGGAGTTTTCTCCAAG
FRD3_BAY CCGTTTTTGTTGGACTAGGTCTCTACTTTGGTGCCGGAATTTTCTCCAAG
FRD3_SHA CCGTTTTTGTTGGACTAGGTCTCTACTTTGGTGCCGGAATTTTCTCCAAG

FRD3_COL GACCCTGCTGTTATTCACCTCATGGCCATCGGAATACCGGTAACTAATAA
FRD3_BAY GACCCTGCTGTTATTCACCTCATGGCCATCGGAATACCGGTAACTAATAA
FRD3_SHA GACCCTGCTGTTATTCACCTCATGGCCATCGGAATACCGGTAACTAATAA

FRD3_COL TCAAATAATAATTACTATAGTATAAAAATCATTTTAAAAGAATTTTACTA
FRD3_BAY TCAAATAATAATTACTATAGTATAAAAATCATTTTAAAAGAATTTTACTA
FRD3_SHA TCAAATAATAATTACTATAGTATAAAAATCATTTTAAAAGAATTTTACTA

FRD3_COL ATGAGAAGAGGTTATATATATTTATGCAGTTTATAGCAGCAACGCAGCCA
FRD3_BAY ATGAGAAGAGGTTATATATATTTATGCAGTTTATAGCAGCAACGCAGCCA
FRD3_SHA ATGAGAAGAGGTTATATATATTTATGCAGTTTATAGCAGCAACGCAGCCA

FRD3_COL ATAAACTCTCTCGCCTTTGTATTGGATGGAGTCAATTTTGGAGCATCTGA
FRD3_BAY ATAAACTCTCTCGCCTTTGTATTGGATGGAGTCAATTTTGGAGCATCTGA
FRD3_SHA ATAAACTCTCTCGCCTTTGTATTGGATGGAGTCAATTTTGGAGCATCTGA

FRD3_COL TTTTGCTTACACTGCATACTCCATGGTATGCACACTATATATACTATGAA
FRD3_BAY TTTTGCTTACACTGCATACTCCATGGTATGCACACTATATATACTATGAA
FRD3_SHA TTTTGCTTACACTGCATACTCCATGGTATGCACACTATATATACTATGAA

FRD3_COL ATGATTAAAATTCCTTTTTTTTTTTTTGAAATGACTTAAACTTTGTCTAT
FRD3_BAY ATGATTAAAATTCCTTCTTTTTTTTTTGAAATGACTTAAACTTTGTCTAT
FRD3_SHA ATGATTAAAATTCCTTTTTTTTTTTT-GAAATGACTTAAACTTTGTCTAT

FRD3_COL CTTTTTTTCTTGTAATCCAATTATGATAAATCAGGTGGGAGTGGCGGCCA
FRD3_BAY CTTTTTTTCTTGTAATCCAATTATGATAAATCAGGTGGGAGTGGCGGCCA
FRD3_SHA CTTTTTTTCTTGTAATCCAATTATGATAAATCAGGTGGGAGTGGCGGCCA

FRD3_COL TAAGCATTGCAGCAGTAATATATATGGCAAAGACCAATGGTTTCATAGGA
FRD3_BAY TAAGCATTGCAGCAGTAATATATATGGCAAAGACCAATGGTTTCATAGGA
FRD3_SHA TAAGCATTGCAGCAGTAATATATATGGCAAAGACCAATGGTTTCATAGGA

FRD3_COL ATATGGATAGCTCTTACAATCTATATGGCTCTCCGGGCTATTACTGGAAT
FRD3_BAY ATATGGATAGCTCTTACAATCTATATGGCTCTCCGGGCTATTACTGGAAT
FRD3_SHA ATATGGATAGCTCTTACAATCTATATGGCTCTCCGGGCTATTACTGGAAT

FRD3_COL TGCCAGGTATTTAAATTGGGCCTT-----------TACTATAGCCCACT
FRD3_BAY TGCCAGGTATTTTTATTGGGTCTTAATGGGCCTTTACATAATAGCCCACT
FRD3_SHA TGCCAGGTATTTAAATTGGGCCTT-----------TACTATAGCCCACT

FRD3_COL ATAGTAGAAGCAGTATTTGACTGAGTGTTTGAATTTATGCAGGATGGCGA
FRD3_BAY ATAGTAGAAGCAGTATTTGACTGAGTGTTTGAATTTATGCAGGATGGCAA
FRD3_SHA ATAGTAGAAGCAGTATTTGACTGAGTGTTTGAATTTATGCAGGATGGCGA

FRD3_COL CAGGAACTGGACCGTGGAGGTTCTTGCGTGGACGATCATCCTCTTCATCT
FRD3_BAY CAGGAACTGGACCTTGGAGGTTCTTGCGTGGACGATCATCCTCTTCATCT
FRD3_SHA CAGGAACTGGACCGTGGAGGTTCTTGCGTGGACGATCATCCTCTTCATCT

FRD3_COL TCCTAGGACTTAGTTTATTTATAACGAGTTGCATCTCTTCTTCCTTCTTC
FRD3_BAY TCCTAGGACTTAGTTTATTTATAACGAGTTGCATCTCTTCTTCCTTCTTC
FRD3_SHA TCCTAGGACTTAGTTTATTTATAACGAGTTGCATCTCTTCTTCCTTCTTC

FRD3_COL GTTTTTGTTTATGGTTCTTGTGTTTGTTTTTCAACATTTTGTTCGAGAGA
FRD3_BAY GTTTTTGTTTATGGTTCTTGTGTTTGTTTTTCAACATTTTGTTCGAGAGA
FRD3_SHA GTTTTTGTTTATGGTTCTTGTGTTTGTTTTTCAACATTTTGTTCGAGAGA

FRD3_COL CCGTTATCATATTATCAGTTTCACATAAATAATGCATATTTTTAAGTCAT
FRD3_BAY CCGTTATCAT--TATCAGTTTCACATAAATAATGCATATTTTTAAGTCAT
FRD3_SHA CCGTTATCATATTATCAGTTTCACATAAATAATGCATATTTTTAAGTCAT

FRD3_COL TAAAATATGGAGCCCTCTGCCCTCACTGGCTTTTCTACCCAAATAGAATA
FRD3_BAY TAATATATGGAGCCCTCTGCCCTCACTGGCTTTTCTACCCAAATAGAATA
FRD3_SHA TAAAATATGGAGCCCTCTGCCCTCACTGGCTTTTCTAACCAAATAGAATA

FRD3_COL TTCTGTAT-GAGTATGTTTCAGCTAGACTAGATGGTTCAAAGATATCCGG
FRD3_BAY TTCTGTATTGAGTATGTTTTAGCTAGACTAGATAGTTCAAAGATATCCGG
FRD3_SHA TTCTGTAT-GAGTATGTTTCAGCTAGACTAGATGGTTCAAAGATATCCGG

FRD3_COL TGTCCAAGTGTCGATTTGGTGTTGGTTAAAAAAAATGTAAGAAAAAAAAG
FRD3_BAY TGTCCAAGTGTCGATTTGGTGTTGGTTAAAAAAAATGTAAGAAAAAAAAG
FRD3_SHA TGTCCAAGTGTCGATTTGGTGTTGGTTAAAAAAA-TGTAAGAAAAAAAAG

FRD3_COL CTGGATCTTATCCTTTTACTAAAGAAAATCTGACCACAGTTATTTCCTTA
FRD3_BAY CTGGATCTTATCCTTTTACTAAAGAAAATCTGACCATAGTTATTTCCTTA
FRD3_SHA CTGGATCTTATCCTTTTACTAAAGAAAATCTGACCATAGTTATTTCCTTA

FRD3_COL GTTAAGAGCCAAAATTAGGCTCAGATTTTAATTAATTGAACCATTAGATT
FRD3_BAY GTTAAGAGCCAAAATTAGGCTCAGATTTTAATTAATTGAACCATTAGATT
FRD3_SHA GTTAAGAGCCAAAATTAGGCTCAGATTTTAATTAATTGAACCATTAGATT

FRD3_COL TGTTCTTACTCAATTTCTATGTATTTTAATAAGTTTCACTTCTAAAAGTG
FRD3_BAY TGTTCTTACTCAATTTCTATGTATTTTAATAAGTTTCACTTCTAAAAGTG
FRD3_SHA TGTTCTTACTCAATTTCTATGTATTTTAATAAGTTTCACTTCTAAAAGTG

FRD3_COL TTTTCTAAAGTTTTTCAACTATGTACTTCAAATCTTAAGACTGTTTTTAA
FRD3_BAY TTTTCTAAAGTTTTACAACTA----CTTCAAATCTTAAGACTGTTTTTAA
FRD3_SHA TTTTCTAAAGTTTTTCAACTATGTACTTCAAATCTTAAGACTGTTTTTAA

FRD3_COL TGAATCCAAAATTTACACTTTAATTCAAATTTTCAACTTGTGATTAACAA
FRD3_BAY TGAATTCAAAATTTACACTTTAATTCAAATTTTCAACTTGTGATTAACAA
FRD3_SHA TGAATCCAAAATTTACACTTTAATTCAAATTTTCAACTTGTGATTAACAA

FRD3_COL ATAATTATACTTTATAGGTATTAATTTCCTTGCACTTTTTGGAATG
FRD3_BAY ATAATTATACTTTATCGGTATTAATTTCCTTGCACTTTTTGGAATG
FRD3_SHA ATAATTATACTTTATAGGTATTAATTTCCTTGCACTTTTTGGA
```

Figure S6. Continued
