# Supplementary material for: Natural Variation at the FRD3 MATE Transporter Locus Reveals Cross-Talk between Fe Homeostasis and Zn Tolerance in Arabidopsis thaliana
Source: PLoS Genet. 2012 Dec 6;8(12):e1003120. doi: 10.1371/journal.pgen.1003120 (PMC3516540; doi:10.1371/journal.pgen.1003120)
Supplement: Table S1 — QTL analyses. (a) Relative variation of the Primary Root length between 150 µM Zn and control condition (RelPR150). (b) The position of the QTL is expressed in cM from the 1st marker of the chromosome. (c) Name of the closest marker, according to the initial mapping approach. (d) Percentage of variance explained by the QTL. (e) Additive effect. Positive value indicates that for the three QTLs, the presence of the Sha allele at QTL decreases root length in the presence of Zn. (PDF) [file pgen.1003120.s014.pdf]

Table S1. QTL analyses

QTL	Trait[a]	Chromosome	QTL position (cM) [b]	Marker[c]	LOD score	R^2 value[d]	a[e]
ZnT1	$RelPR_{150}$	1	55.6	F5I14	5.99	12	3.66
ZnT2	$RelPR_{150}$	3	6.0	ATHCHIB2	14.1	29	5.58
ZnT3	$RelPR_{150}$	5	51.2	MSAT5.9	6.94	16	4.13

a. Relative variation of the Primary Root length between 150 µM Zn and control condition ($RelPR_{150}$)
b. The position of the QTL is expressed in cM from the 1st marker of the chromosome
c. Name of the closest marker, according to the initial mapping approach
d. Percentage of variance explained by the QTL
e. Additive effect. Positive value indicates that for the three QTLs, the presence of the Sha allele at QTL decreases root length in the presence of Zn.
