# Supplementary material for: Natural Variation at the FRD3 MATE Transporter Locus Reveals Cross-Talk between Fe Homeostasis and Zn Tolerance in Arabidopsis thaliana
Source: PLoS Genet. 2012 Dec 6;8(12):e1003120. doi: 10.1371/journal.pgen.1003120 (PMC3516540; doi:10.1371/journal.pgen.1003120)
Supplement: Table S2 — Haplotypes and phenotypes of A. thaliana accessions genotyped at FRD3. (a) Representative haplotype according to Figure S7. (b) Inhibitory concentration 50 (µM) determined as the Zn concentration that reduced primary root length to 50% of control. Stars refer to data from reference [16]. (c) Accession number according to INRA Versailles Genomic Resource Centre (http://dbsgap.versailles.inra.fr/vnat/). (d) Accession number according to NASC, European Arabidopsis Stock Centre (http://arabidopsis.info). (PDF) [file pgen.1003120.s015.pdf]

Table S2. Haplotypes and phenotypes of *A. thaliana* accessions genotyped at *FRD3*

Name	Haplotype[a]	IC50[b]	AV code[c]	NASC code[d]	Name	Haplotype	IC50	AV code	NASC code
9481B	Sha	191	261AV	CS22460	Mh-0	Col		175AV	N904
Abd-0	Ita		177AV	N932	Mh-1	Col	193*	215AV	N1368
Akita	Ita	189*	252AV	-	Mir-0	Col		216AV	N1378
Alc-0	Cvi	142*	178AV	N1656	Ms-0	Ita		93AV	N905
Altaï-2	Cvi		547AV	-	Mt-0	Ita	144*	94AV	N1380
Altai-5	Cvi		548AV	-	N10	Ita		265AV	CS22488
An-1	Ita		96AV	N944	N13	Col	146*	266AV	CS22491
Anz-1a	Col		705AV		N14	Col		267AV	CS22492
Bay-0	Bay	165*	41AV	N954	N15	Ita		268AV	CS22493
Bik-1	Col		541AV	-	N4	Col		260AV	CS22482
Bik-7	Cvi		543AV	-	N6	Ita		262AV	CS22484
Bl-1	Ita	128*	42AV	N968	N7	Col		263AV	CS22485
Bla-1	Ita		76AV	N970	N8	Ita		264AV	CS22486
Blh-1	Cvi	159*	180AV	N1030	Nd-1	Bay		220AV	N1636
Br-0	Ita		60AV	N994	Neo-3	Sha		539AV	-
Bur-0	Col	195*	172AV	N1028	Néo-6	Sha		540AV	-
C24	Ita		183AV	N906	Niigata	Ita		255AV	-
Can-0	Bay	170*	163AV	N1064	No-0	Bay		49AV	N3081
Chi-0	Ita		89AV	N1072	Ost-0	Col		61AV	N1430
Co-4	Bay		174AV	N1090	Oy-0	Col	166*	224AV	N1436
Col-0	Col	193*	186AV	N1092	Pa-1	Ita		50AV	N1438
Ct-1	Bay	155*	162AV	N1094	Per-1	Ita		100AV	N1444
Cvi-0	Cvi	149*	166AV	N902	Petergof	Ita		226AV	N926
Db-1	Col		132AV	N1102	Pi-0	Ita		40AV	N1454
Dja-1	Cvi		534AV	-	Pyl-1	Ita	206*	8AV	-
Dja-5	Cvi		535AV	-	Qar-8a	Col		549AV	-
Dr-0	Ita		51AV	N1114	Rak-2	Ita		228AV	N1484
Dra-0	Col		194AV	N1116	Ran	Col		21AV	-
Edi-0	Bay	154*	83AV	N1122	Ri-0	Ita		160AV	N1492
Enkheim-T	Ita		197AV	N921	Rld-2	Col		229AV	N1641
Est-0	Ita		71AV	N1148	Rsch-4	Ita		90AV	N1494
Fl-1	Col		199AV	N1160	Rubezhnoe-1	Cvi	137	231AV	N927
Ge-0	Ita		101AV	N1186	Sah-0	Ita		233AV	N1500
Gr-3	Col		58AV	N1202	Sakata	Ita	190*	257AV	-
Gre-0	Bay	154*	200AV	N1210	Sap-0	Bay		234AV	N1506
Had-1b	Cvi		544AV	-	Sav-0	Ita		235AV	N1514
Hiroshima	Sha	187	254AV	-	Shahdara	Sha	136*	236AV	N929
Hodja-Obi-Garm	Cvi	128	203AV	CS922	Sorbo	Ita		238AV	CS931
Ishikawa	Sha	133	253AV	-	Sp-0	Col		53AV	N1530
Ita-0	Ita	151*	157AV	N1244	St-0	Bay	154*	62AV	N1534
Jea	Col	166*	25AV	-	Stw-0	Sha	139*	92AV	N1538
Jl-3	Ita		205AV	N1252	Sus-1	Cvi		533AV	-
Jm-0	Col		206AV	N1258	Ta-0	Col		56AV	N1548
Ka-0	Col		55AV	N1266	Te-0	Ita		68AV	N1550
Kar-1	Cvi		531AV	-	Tokushima	Ita		258AV	-
Kar-2	Cvi		532AV	-	Tsu-0	Ita	128*	91AV	N1564
Kas-1	Ita		108AV	N903	WestKar-3	Cvi		709AV	
Kin-0	Ita		209AV	N1272	WestKar-4	Cvi		710AV	
Kn-0	Ita	145*	70AV	N1286	Wil-1	Col		72AV	N1594
Ko-2	Bay		46AV	N1288	Ws	Ita		244AV	-
Kondara	Cvi	158	190AV	N916	Ws-0	Bay		84AV	N915
Kyr-1	Cvi		538AV	-	Yo-0	Ita		250AV	N1622
Ldv-5	Col		11AV	-	Zal-1	Cvi		536AV	-
Ler-1	Ita		213AV	N1642	Zal-3	Cvi		537AV	-
Lip-0	Ita		63AV	N1336					

a. Representative haplotype according to Figure S7
b. Inhibitory concentration 50 (μM) determined as the Zn concentration that reduced primary root length to 50% of control. Stars refer to data from reference [16].
c. Accession number according to INRA Versailles Genomic Resource Centre (http://dbsgap.versailles.inra.fr/vnat/)
d. Accession number according to NASC, European Arabidopsis Stock Centre (http://arabidopsis.info)
