# Supplementary material for: Natural Variation at the FRD3 MATE Transporter Locus Reveals Cross-Talk between Fe Homeostasis and Zn Tolerance in Arabidopsis thaliana
Source: PLoS Genet. 2012 Dec 6;8(12):e1003120. doi: 10.1371/journal.pgen.1003120 (PMC3516540; doi:10.1371/journal.pgen.1003120)
Supplement: Table S3 — Segregation analysis of the Fe overaccumulation trait in the F2 plants issuing from a cross between Shahdara and frd3-7. (a) Genotype determined for the presence or absence of T-DNA using primers LBb1 and FRD3exon12R or FRD3exon7F and FRD3exon12R, respectively. (b) Fe overaccumulation is visualized by Perls' stain [21] of intact roots of plants grown on compost for 3 weeks. (c) Chi2 analysis of the segregation of Fe overaccumulation trait in the F2 progeny, H0 hypothesis was that this trait is under the control of one monogenic recessive locus. (PDF) [file pgen.1003120.s016.pdf]

Table S3. Segregation analysis of the Fe overaccumulation trait in the F2 plants issuing from a cross between Shahdara and *frd3-7*.

Genotype[a]	Number of plants showing		χ_2 test[c]
	Fe overaccumulation in stele [b]	No Fe overaccumulation in stele	
All F2 plants	14	44	0.02
frd3/*frd3*	9	2	
frd3/FRD3^{Sha}	5	34	
FRD3^{Sha}/FRD3^{Sha}	0	8	

a. Genotype determined for the presence or absence of T-DNA using primers LBb1 and FRD3exon12R or FRD3exon7F and FRD3exon12R, respectively.
b. Fe overaccumulation is visualized by Perls' stain [21] of intact roots of plants grown on compost for 3 weeks.
c. Chi_2 analysis of the segregation of Fe overaccumulation trait in the F2 progeny, H0 hypothesis was that this trait is under the control of one monogenic recessive locus.
