# Supplementary material for: Natural Variation at the FRD3 MATE Transporter Locus Reveals Cross-Talk between Fe Homeostasis and Zn Tolerance in Arabidopsis thaliana
Source: PLoS Genet. 2012 Dec 6;8(12):e1003120. doi: 10.1371/journal.pgen.1003120 (PMC3516540; doi:10.1371/journal.pgen.1003120)
Supplement: Table S4 — List of markers. (a) Position on chromosome 3 in bp. (b) CAPS for Cleaved Amplified Polymorphic Sequence; INDEL for insertion or deletion; MSAT for microsatellite; SNP for Single Nucleotide Polymorphism. (c) RE for Restriction Enzyme used for CAPS markers. (d) Markers used for the haplotyping of AtFRD3 in accessions of A. thaliana. (PDF) [file pgen.1003120.s017.pdf]

Table S4. List of markers

Name	Position[a]	Type[b]	RE[c]	Forward (5'-3')	Reverse (5'-3')
NGA172	786 400	MSAT		AGCTGCTTCCTTATAGCGTCC	CATCCGAATGCCATTGTTC
MSAT302422	2 422 445	MSAT		CGGAATTAAGAACCGAATTGA	ACAGGAAGGCTTCTGGGAAT
MSAT302503	2 503 754	MSAT		TTGTTGATTGCCCCATTTTT	AAGGAATGAAATGATACATTTGGAA
CAPS2524188	2 524 188	CAPS	TaqI	CAACTCCGGCAGTGATTTTG	AACCAATGACGTCTTTCCAGA
CAPS2533212	2 533 212	CAPS	StuI	TGCTTCCCTGCTCGTTAAGT	TGGGAAGATATGCTGCTGAA
CAPS2557387	2 557 387	CAPS	Hpy188I	TGAAGCTGAAGCTTGGGAAT	TACCGCAAAAGCAAAAGAGG
CAPS2560983	2 560 983	CAPS	MnlI	CAACTCCATGAACTCCAATGAA	GATGAATTGGCGGAGGAAG
CAPS2564930	2 564 930	CAPS	HinfI	ACGCTGTCGCCATTAAAGAG	GAACCAAAGTGAACCAAAAACA
IND302567 in12[d]	2 566 751	INDEL		ATATGGCTCTCCGGGCTATT	ATGATCGTCCACGCAAGAAC
CAPS302569[d]	2 568 974	CAPS	BsrI	AGCTTTGGCTTTAGCTGCTG	CGGAATTTACCTGGAGGTTG
IND302570 del27[d]	2 569 837	INDEL		CGAGTAAAAGTTCACGAAAACCA	CCGTCCAAACTTAAGACACCA
IND302570 del28[d]	2 570 150	INDEL		TTCTATACCAAATTAACCCGAAAA	TTCTCATTTTTCTTTGATATTTTCCA
SNP2579477	2 579 477	SNP		TGTTCGACAAGTCTACCATGAAA	TTCTGTTTGTTGGTCAACGATT
CAPS2587730	2 587 730	CAPS	RsaI	GGCAACATTTGGGCATACAT	GGCTCTAGAACTTGCCTGGA
CAPS2619300	2 619 300	CAPS	AluI	ATGTGGTGCCATCTGTAGCC	ACCCGTAGTTGCTTCCACAC
CER464947	3 109 833	MSAT		CTCACCTTGCTGGAGTCCG	GTAAAGGTTTAGGCAGAGTGTG
ATHCHIB2	3 963 350	MSAT		GGATCCAAGTGCTCATATATAC	CTTTCGTTTCTAAATATGAGAAGC
CAPS4942156	4 942 156	CAPS	HinfI	TGGAAGCTCTAGAAACGATCG	ACCACCTAAACCGAGAATTGG
CAPS7012599	7 125 259	CAPS	ClaI	CACCTAACAGTTCACACCAA	CCTTGATTTCCCTACAACAG
MSAT3.19	8 808 167	MSAT		TAATTCGATCCAATTGACAT	TGGCTTGGCACAAAC

a. Position on chromosome 3 in bp.
b. CAPS for Cleaved Amplified Polymorphic Sequence; INDEL for insertion or deletion; MSAT for microsatellite; SNP for Single Nucleotide Polymorphism
c. RE for Restriction Enzyme used for CAPS markers
d. Markers used for the haplotyping of *AtFRD3* in accessions of *A. thaliana*
