# Supplementary material for: Natural Variation at the FRD3 MATE Transporter Locus Reveals Cross-Talk between Fe Homeostasis and Zn Tolerance in Arabidopsis thaliana
Source: PLoS Genet. 2012 Dec 6;8(12):e1003120. doi: 10.1371/journal.pgen.1003120 (PMC3516540; doi:10.1371/journal.pgen.1003120)
Supplement: Table S5 — List of primers. (a) Present study. (b) ASE for Allele-Specific Expression. (PDF) [file pgen.1003120.s018.pdf]

Table S5. List of primers

Application	Primer name	Forward (5'-3')	Reverse (5'-3')	Ref.
seq. & cloning	FRD3_promo1F	TGACGTTGATGGCTTGAAAA		a
sequencing	FRD3_promo2F	TTTGGACGGAAATAAAGAGCA		a
sequencing	FRD3_exon 2F	GGCAGAGGAAGACACGATGG		a
sequencing	FRD3_intron 4F	GCCTTAATGCGTGAAACAGTC		a
sequencing	FRD3_exon 12R		TTCCTGTTGCCATCCTGCAT	a
seq. & cloning	FRD3_post3'R		CATTCCAAAAAGTGCAAGGAA	a
cDNA modification	FRD3-V417I	TCCTTGGAGAAAATTCCGGCACCAA	TTGGTGCCGGAATTTTCTCCAAGGA	a
cDNA modification	FRD3-LP-NS	TTTCTGCATGAACAGGACTGGCTTTGTTTGCT	AGCAAACAAAGCCAGTCCTGTTCATGCAGAAA	a
cDNA modification	FRD3-L117P	ACAAAGCCAATCCTGTTCATGCAGA	TCTGCATGAACAGGATTGGCTTTGT	a
cDNA modification	FRD3-N116S	GCATGAACAAGACTGGCTTTGTTTG	CAAACAAAGCCAGTCTTGTTCATGC	a
Q-RTPCR	AtIRT1 (At4g19690)	CGGTTGGACTTCTAAATGC	CGATAATCGACATTCCACCG	36
Q-RTPCR	AtFRO2 (At1g01580)	GCGACTTGTAGTGCGGCTATG	CGTTGCACGAGCGATTCTTG	36
Q-RTPCR	AtFRD3 (At3g08040)	CGAGGACCATAGCAGTGACG	CAGCAAACGAACAAGCCAGA	a
Q-RTPCR	Clathrin (At4g24550)	AGCATACACTGCGTGCAAAG	TCGCCTGTGTCACATATCTC	36
Q-RTPCR	ACT2/ACT8 (At3g18780/At1g49240)	GGTAACATTGTGCTCAGTGGTGG	AACGACCTTAATCTTCATGCTGC	35
Q-RTPCR	PP2A (At1g13320)	TAACGTGGCCAAAATGATGC	GTTCTCCACAACCGCTTGGT	37
Q-RTPCR	At5g12240	AGCGGCTGCTGAGAAGAAGGT	TCTCGAAAGCCTTGCAAAATCT	37
ASE[b]	PyroSNP1-2	TGCTGTGGCTGGTTGGTAT	GGAAGACACGATGGAGAAGATG	a
ASE[b]	PyroSNP1-2_Seq	CAAGTATAGTTTCTGCATGA		a
ASE[b]	PyroSNP3	TGCTGTGGCTGGTTGGTAT	GGAAGACACGATGGAGAAGATG	a
ASE[b]	PyroSNP3_Seq	GCATTTCTTCACCTACAAGT		a
ASE[b]	PyroSNP4	CCCAAAGCTCGTATGCTCAAGT	GCCAATCTTGTTCATGCAGAAACT	a
ASE[b]	PyroSNP4_Seq	CGCCTAGAAGCAACTT		a
T-DNA testing	LBb1	GCGTGGACCGCTTGCTGCAACT		
T-DNA testing	FRD3_exon 12R		TTCCTGTTGCCATCCTGCAT	a
T-DNA testing	FRD3_exon 7F	CGAGGACCATAGCAGTGACG		a

a. present study
b. ASE for Allele-Specific Expression
